# Photosystem II Function and Dynamics in Three Widely Used *Arabidopsis thaliana* Accessions

**DOI:** 10.1371/journal.pone.0046206

**Published:** 2012-09-28

**Authors:** Lan Yin, Rikard Fristedt, Andrei Herdean, Katalin Solymosi, Martine Bertrand, Mats X. Andersson, Fikret Mamedov, Alexander V. Vener, Benoît Schoefs, Cornelia Spetea

**Affiliations:** 1 Department of Biological and Environmental Sciences, University of Gothenburg, Gothenburg, Sweden; 2 Department of Clinical and Experimental Medicine, Linköping University, Linköping, Sweden; 3 Department of Plant Anatomy, Eötvös University, Budapest, Hungary; 4 National Institute for Marine Sciences and Techniques, Cnam, Cherbourg-Octeville, France; 5 Department of Chemistry - Ångström Laboratory, Uppsala University, Uppsala, Sweden; 6 Mer Molécules Santé, EA2160, LUNAM Université, Université du Maine à Le Mans, Le Mans, France; Lawrence Berkeley National Laboratory, United States of America

## Abstract

Columbia-0 (Col-0), Wassilewskija-4 (Ws-4), and Landsberg erecta-0 (L*er*-0) are used as background lines for many public *Arabidopsis* mutant collections, and for investigation in laboratory conditions of plant processes, including photosynthesis and response to high-intensity light (HL). The photosystem II (PSII) complex is sensitive to HL and requires repair to sustain its function. PSII repair is a multistep process controlled by numerous factors, including protein phosphorylation and thylakoid membrane stacking. Here we have characterized the function and dynamics of PSII complex under growth-light and HL conditions. Ws-4 displayed 30% more thylakoid lipids *per* chlorophyll and 40% less chlorophyll *per* carotenoid than Col-0 and L*er*-0. There were no large differences in thylakoid stacking, photoprotection and relative levels of photosynthetic complexes among the three accessions. An increased efficiency of PSII closure was found in Ws-4 following illumination with saturation flashes or continuous light. Phosphorylation of the PSII D1/D2 proteins was reduced by 50% in Ws-4 as compared to Col-0 and L*er*-0. An increase in abundance of the responsible STN8 kinase in response to HL treatment was found in all three accessions, but Ws-4 displayed 50% lower levels than Col-0 and L*er*-0. Despite this, the HL treatment caused in Ws-4 the lagest extent of PSII inactivation, disassembly, D1 protein degradation, and the largest decrease in the size of stacked thylakoids. The dilution of chlorophyll-protein complexes with additional lipids and carotenoids in Ws-4 may represent a mechanism to facilitate lateral protein traffic in the membrane, thus compensating for the lack of a full complement of STN8 kinase. Nevertheless, additional PSII damage occurs in Ws-4, which exceeds the D1 protein synthesis capacity, thus leading to enhanced photoinhibition. Our findings are valuable for selection of appropriate background line for PSII characterization in *Arabidopsis* mutants, and also provide the first insights into natural variation of PSII protein phosphorylation.

## Introduction

Chloroplasts are plant organelles performing a unique and complex process named oxygenic photosynthesis, on which aerobic life depends. The light-dependent reactions of this process take place in the thylakoid membrane, and use sunlight to extract and transfer electrons from water to NADP^+^ through three multisubunit chlorophyll-protein complexes, namely, photosystem II (PSII), cytochrome b_6_f and PSI. A fourth complex is the ATP synthase, which generates ATP by using the electrochemical proton gradient generated across the thylakoid membrane during electron transport.

The water-oxidizing PSII complex in plants is a supercomplex located exclusively in the grana stacks of the thylakoid membrane. This complex consists of a dimeric core with over 25 subunits *per* monomer and of a trimeric outer light-harvesting antenna (LHCII), connected to the core *via* a monomeric inner antenna. Among photosynthetic complexes, PSII is the main target for inactivation by high-intensity light (HL) alone or in combination with other stress factors, a process known as photoinhibition. To survive, plants have evolved a battery of photoprotective short- and long-term mechanisms, including chloroplast avoidance movement, thermal dissipation of excess light energy *via* xanthophyll cycle, phosphorylation of LHCII proteins and nuclear gene expression (for a review, see ref. [1]). Nevertheless, inactivation of PSII still occurs in nature and the reaction center D1-subunit is oxidatively damaged. To replace the damaged subunit, PSII undergoes a multistep repair cycle. This involves reversible phosphorylation of PSII core proteins (D1, D2, CP43 and PsbH) in the grana stacks, PSII monomerization, migration to the non-appressed (stroma) thylakoid regions and partial disassembly, to allow degradation of the damaged D1 and insertion of a new copy (for reviews, see refs. [2], [3]). To facilitate lateral protein mobility, dynamic changes in the stacking of the thylakoid membrane take place [4], [5]. In addition, stacking of thylakoids is important for several other processes, including biogenesis of photosynthetic complexes, regulation of light harvesting and thermal dissipation of excess light energy [6].

Sequencing of *Arabidopsis thaliana* (*Arabidopsis*) genome in the year 2000 allowed an explosion of genomic and proteomic information about chloroplast function and regulation, and also started to reveal the signaling and regulatory components of the PSII repair cycle (for a review, see ref. [7]). Phenotypic analyses of mutants have played a major role in this respect. These mutants often come from public collections generated using several background lines of *Arabidopsis*, and are characterized in comparison with the respective background line in laboratory conditions. Less attention has been paid to analyzing differences in development, growth and stress response among the accessions, although it could be valuable for the selection of an appropriate background line for mutant characterization.

Naturally occurring genetic variation is extensively studied in *Arabidopsis* (for reviews, see refs. [8], [9]). When it comes to natural genetic variation in plant photosynthesis, so far only two traits have been investigated in *Arabidopsis*, namely thermal dissipation of excess light energy [10] and the small subunit of Rubisco [11]. For other plant species (*e.g.,* rice, maize, wheat), the investigated photosynthetic traits have been chlorophyll (Chl) content, Chl fluorescence, photosynthetic rate *per* unit leaf area, and tolerance to HL and cold (for a review, see ref. [12]).

In this article, the function and dynamics of PSII complex were studied in three *Arabidopsis* accessions, which are widely used as background lines for generation and characterization of mutants (TAIR, http://www.arabidopsis.org/) in laboratory conditions. Columbia-0 (Col-0) is a background line for SALK T-DNA mutants, Wassilewskija-4 (Ws-4) for INRA Versailles T-DNA lines, and Landsberg *erecta*-0 (L*er*-0) for JIC Gene trap Ds lines. They are also named lab accessions since they are standard genotypes, which have been propagated under laboratory conditions for at least 60 years, although originally descendant from wild collected genotypes. The precise location of collection has been lost or mixed up over the years. In this study we report that Ws-4 is the most susceptible to HL, and explain this based on a lipid-Chl-carotenoid stoichiometry in the thylakoid membrane, which is significantly distinct from the one in the other two accessions. We also found that STN8-catalyzed PSII D1/D2 protein phosphorylation is a component of HL acclimation strategy, which is working at 50% levels in Ws-4 as compared to Col-0 and L*er*-0.

## Results

### Plant growth and PSII function under GL conditions

**Figure 1:**
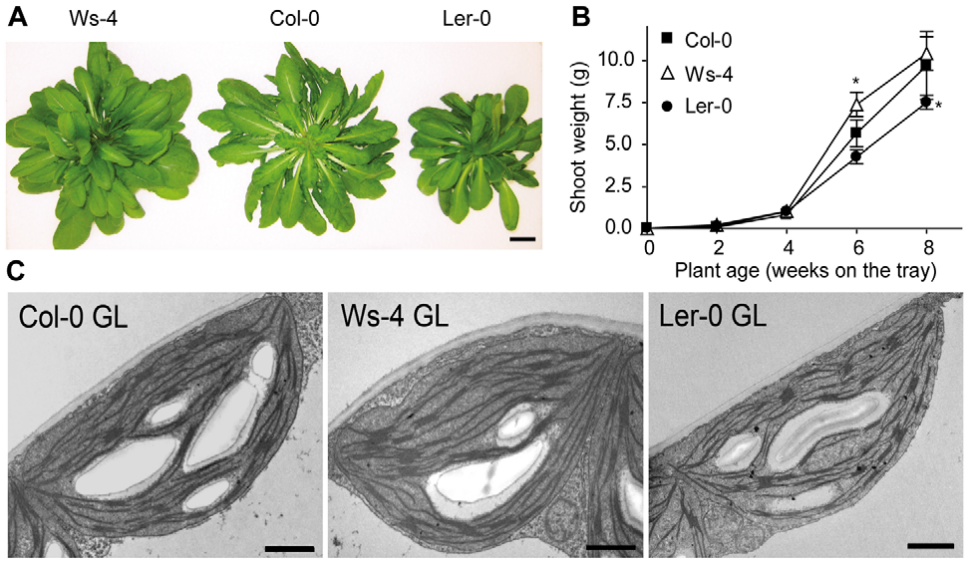
Growth and chloroplast ultrastructure of Col-0, Ws-4 and L*er*-0 *Arabidopsis* accessions. Plants were grown using hydroponic system at an irradiance of 120 µmol photons m^−2^ s^−1^ (GL). (A) Photographs of representative plants grown for 6 weeks. Scale bar: 2 cm. (B) Plot of shoot weight ±SD as a function of plant age (n = 10). Asterisks indicate significant difference at Student's t-test P<0.01. SD bars are shown when larger than the symbols. (C) Representative transmission electron micrographs of plants grown for 4 weeks. Scale bar: 1 µm.

The activity of PSII has been initially characterized in Col-0 and Ws-4, previously used as background lines during phenotypic analyses of *Arabidopsis* mutants lacking the thylakoid ATP/ADP carrier [13]. A third selected *Arabidopsis* accession for the present study was L*er*-0. All three accessions displayed distinct visual phenotype in terms of rosette and individual leaf shape when grown either hydroponically or on soil (Figures 1A and S1), as described at TAIR site. The most remarkable feature also documented at TAIR is the lighter green color of Ws-4 leaves. The shoot fresh weight in the first four weeks of hydroponic cultivation at irradiance of 120 µmol photons m^−2^ s^−1^ (GL) was found similar for the three accessions (Figure 1B). However, when reaching full development, at an age of about six weeks, the fresh weights of Ws-4 and L*er*-0 were approx. 30% higher and 25% lower, respectively, than that of Col-0 (Figure 1B; Table 1). The dry weight of the shoots was also found to differ among the accessions, namely 0.46±0.06 g for Col-0 (100%), 0.53±0.05 g for Ws-4 (115%) and 0.41±0.08 g for L*er*-0 (89%). When grown on soil for six weeks, the plants were smaller and had a 3–5 fold lower shoot weight than the plants at the same age grown hydroponically (Figure S1; Table S1). Moreover, only L*er*-0 had 15% lower fresh weight when grown on soil, whereas Ws-4 and Col-0 displayed similar shoot weight. The reason for a higher shoot weight for Ws-4 grown hydroponically is unclear. Nevertheless, the Ws-4 leaves were lighter green than the leaves of the other two accessions under both types of cultivation conditions.

**Table 1:** Leaf biomass, chlorophyll content and PSI to PSII ratio of Col-0, Ws-4, and L*er*-0 accessions.

Accession	Shoot weight	Leaf specific mass	Leaf chlorophyll content^a^			PSI/PSII^b^
	*g plant^−1^*(n = 11–18)	*mg cm^−2^*(n = 8–15)	*µg cm^−2^*(n = 8–15)	*mg g^−1^*(n = 8–15)	*Chl a/b*(n = 8–15)	(n = 3)
Col-0	5.72±0.69100%	16.77±1.18100	49.84±5.34100%	2.97±0.12100%	4.00±0.26	1.06±0.09
Ws-4	7.38±0.68*130%	13.32±0.47*79%	41.19±4.79*83%	3.09±0.17104%	3.80±0.04	1.11±0.02
L*er*-0	4.42±0.83*77%	17.39±1.90103%	53.68±3.37108%	3.08±0.28103%	3.86±0.29	1.12±0.08

a Leaf chlorophyll content was measured spectrophotometrically after extraction in ethanol, and expressed on leaf area and on fresh weight basis.

b PSI/PSII ratio was measured by EPR spectroscopy in isolated thylakoids.

The data were expressed as means ±SD (n = number of replicates). The parameters were also expressed relative to Col-0. *, Significantly different from Col-0 (Student's t-test P<0.05).

The parameters were measured on plants grown hydroponically for six weeks at an irradiance of 120 µmol photons m^−2^ s^−1^.

Chl was extracted from leaves detached from plants grown for six weeks under GL conditions either hydroponically or on soil. Under both types of cultivation conditions, the Chl content when expressed *per* unit leaf area was found significantly lower in Ws-4 (by approx. 20%) than in Col-0 and L*er*-0 (Tables 1 and S1). Also under both types of cultivation conditions, the leaf specific mass of Ws-4 was found lower than for the other two accessions. Thus, when expressed *per* leaf weight, the Chl content was found similar in the three accessions. Furthermore, there was no significant difference among the studied accessions either in the Chl *a/b* ratio, which is an indicator for the ratio of PSII cores to LHCII, or in the PSI/PSII ratio (Tables 1 and S1). Similarly, no large difference was observed by electron microscopy in the chloroplast ultrastructure (Figure 1C; Table S2). Except for the difference in shoot weight of Ws-4, the three accessions displayed similar pattern in all Chl-related parameters when grown either hydroponically or on soil. Therefore, for biochemical experiments requiring larger amounts of leaf material, we have used plants grown hydroponically, whereas for other types of experiments plants grown either on soil or hydroponically were used, as indicated in Materials and Methods and Legends to Figures.

**Figure 2:**
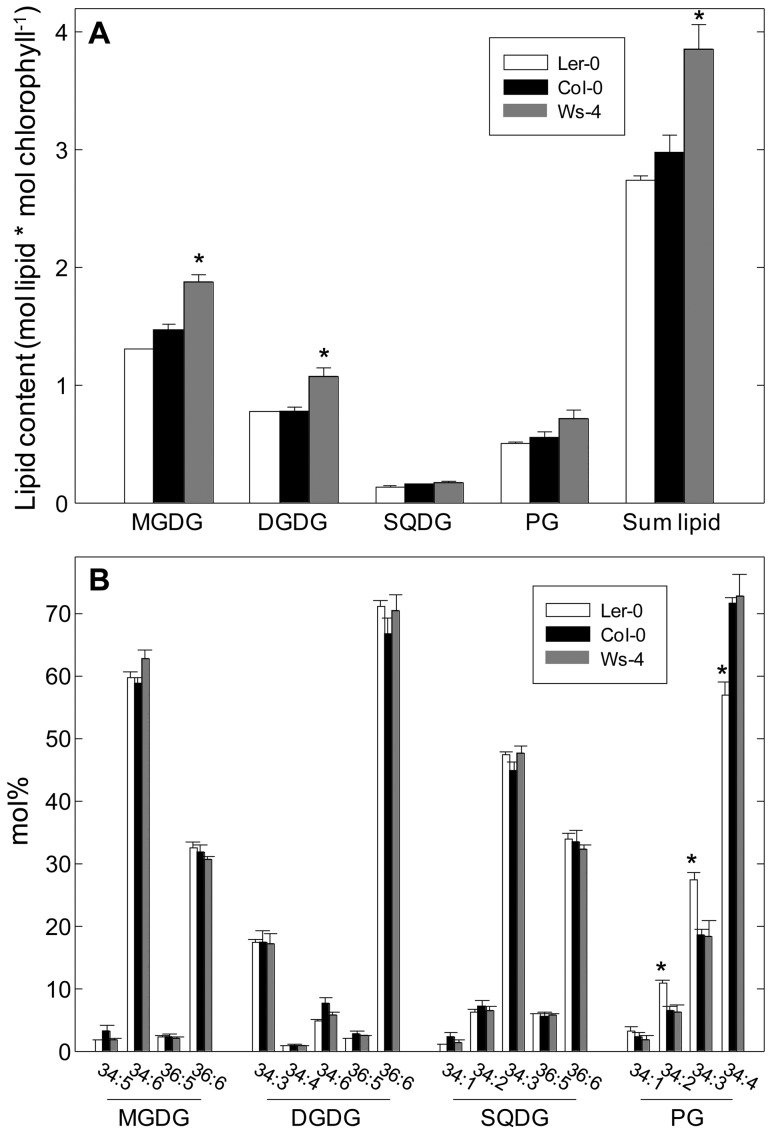
Thylakoid lipid composition in Col-0, Ws-4 and L*er*-0 accessions. Membrane lipids were extracted from plants grown on soil for six weeks and analyzed by LC-MS. (A) The content of each lipid class was expressed as a molar ratio to chlorophyll. (B) Lipid species comprising more than 2% of the lipid class are shown as mol % of the total lipid class. Numbers indicate total number of carbons and double bonds in lipid fatty acids. The plotted data represent means ±SD (n = 3). An asterisk indicates significant difference from Col-0 (Student's t-test P<0.05).

Lipids play an important role in organizing Chl-protein complexes in the thylakoid membrane [14]. Therefore, we have analyzed the abundance and composition in leaves of four thylakoid lipid classes, namely monogalactosyldiacylglycerol (MGDG), digalactosyldiacylglycerol (DGDG), sulfoquinovosyldiacylglycerol (SQDG), and phosphatidylglycerol (PG). Although the latter is not only present in thylakoids, the bulk of PG in a leaf is expected to be associated with the thylakoid membrane. The overall lipid composition for Col-0 (Figure 2A; Table S3) was found in line with previous reports [15]. Notably, Ws-4 contained a significantly higher amount of thylakoid lipids than the other two accessions when expressed on a leaf fresh weight basis (Table S3). This difference was compensated by the lower leaf specific mass of Ws-4 (Table S1), so that all three accessions had similar amounts of lipids when expressed on a leaf area basis. Nevertheless, the lipid-to-Chl molar ratio was found approx. 3.75 in Ws-4 and 3.0 in the other two accessions, *i.e.* about 30% higher in Ws-4, mainly due to a significantly higher MGDG and DGDG content relative to Chl (Figure 2A). The observation of an increase in MGDG-Chl ratio is interesting, since MGDG represents a non-bilayer lipid exerting lateral membrane pressure and modulating the PSII array formation [14], [16]. The lipid species composition of the thylakoid membrane differed only with respect to PG species since L*er*-0 contained approx. 20% less 34∶4-PG than the other two accessions (Figure 2B). This was compensated by an increased proportion of 34∶2- and 34∶3-PG in L*er*-0. Based on the MRM method used (neutral loss of a head group specific fragment), it is not possible to discern the exact fatty acid composition of these species. Product ion scan in negative mode of the three PG species, however, revealed that the detected 34∶4 contained 16∶1 and 18∶3, whereas 34∶2 and 34∶3 contained 16∶0 and 18∶2 or 18∶3, respectively. Thus, thylakoid PG in L*er*-0 apparently contained approx. 20% less 16∶1 than it did in the other two accessions. The role of 16∶1-PG species is not clearly understood in *Arabidopsis,* since mutants deficient in this lipid species did not display any physiological phenotype, performed normal energy transfer from LHC to PS, and the stability of the complexes was not found affected [17], [18]. Taken together, the lipid composition is similar in the three analyzed accessions, but additional MGDG and DGDG dilute the Chl-protein complexes in the thylakoid membrane of Ws-4.

To determine the maximum quantum yield of PSII photochemistry, kinetics of PSII closure (Q_A_ reduction) were recorded using Chl fluorescence in leaves from dark-adapted plants. The shape of the O-J-I-P fluorescence induction curves (Figure S2), and the values obtained for the *F*_v_/*F*_m_ (0.80) and the relative variable fluorescence at the J-step (V_J_) between 0.36 and 0.47, are typical for healthy plants [19]. The initial slope of the relative variable fluorescence curve (M_0_) indicates the net rate of closure of PSII reaction centers (RCII), whereas M_0_/V_J_ represents the maximum rate of RCII closure [20]. The values for both parameters were found slightly (5%) but significantly higher in Ws-4 (Table 2), indicating that RCII closed faster than in the other two accessions.

**Table 2:** Fundamental parameters of the O-J-I-P fluorescence induction curves recorded on attached leaves of Col-0, Ws-4 and L*er*-0 accessions.

Parameter	Col-0	Ws-4	L*er*-0
*F*_v_/*F*_m_	0.805±0.003	0.808±0.012	0.802±0.009
M_0_	1.650±0.030	1.711±0.044*	1.574±0.151
M_0_/V_J_	3.796±0.064	3.982±0.081*	3.761±0.125

The significant differences are indicated by asterisk (Student's t test P<0.05).

The plants were grown on soil for 15–17 days at an irradiance of 120 µmol photons m^−2^ s^−1^. The leaves were adapted to darkness for 15 min before the measurement (n = 20–25).

**Figure 3:**
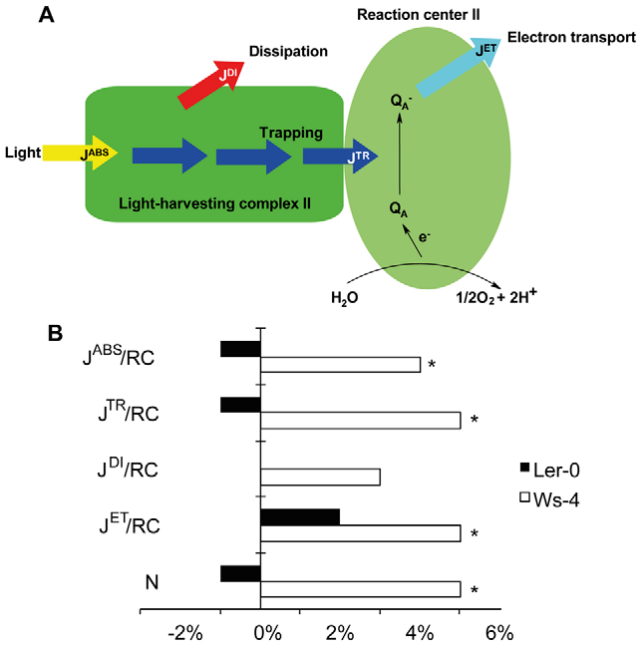
J fluxes per reaction center in Col-0, Ws-4 and L*er*-0 accessions. (A) A highly simplified model of the various types of J fluxes per reaction center of photosystem II (RCII). J^ABS^ is the total photon flux absorbed by the RCII. J^DI^ is the flux of energy that is dissipated in the light-harvesting complex II as heat and/or fluorescence. J^TR^ is the flux of energy used for trapping in the RCII. J^ET^ is the electron flux between the two photosystems. (B) Plot of J fluxes per RC. N is the number of electrons in the transport chain. The J fluxes *per* RC were calculated, the values obtained were subtracted from those obtained for Col-0, and then expressed relatively to Col-0. Asterisks indicate significant difference at P<0.05 (Student's t-test, n = 20–25).

Based on the same O-J-I-P fluorescence induction curves, it was next determined whether RCII of the three accessions were managing similarly or not the incident photons during a saturation pulse. This includes determination of the total photon flux absorbed *per* RC (J^ABS^, formerly ABS parameter), the proportion of this flux that is either dissipated in the LHCII antenna as heat and/or fluorescence (J^DI^, formerly parameter DI_0_) or used for trapping in the RCII (J^TR^, formerly parameter TR_0_) [20]. A simplified model of the J fluxes is presented in Figure 3A. Within RCII, the energy is converted to redox energy by reducing the first stable quinone electron acceptor Q_A_, while RCII Chl P_680_ is oxidized, subsequently creating an electron flux between PSII and the PSI electron acceptor ferredoxin within the photosynthetic apparatus (J^ET^, formerly parameter ET_0_). As defined, the J fluxes are interrelated and are dependent on the structural property and photosynthetic activity of the samples. To understand the meaning of this analysis it is important to keep in mind that the J parameters characterize the Q_A_-reducing PSII when working at their maximum capacity.

The J fluxes *per* RC were calculated, the values obtained were subtracted from those obtained for Col-0, and then expressed relatively to Col-0. The obtained values for L*er*-0 did not differ significantly from those obtained for Col-0 (0%), whereas the values for J^ABS^/RC, J^TR^/RC and J^ET^/RC were significantly higher in Ws-4 (Figure 3B). A 4% higher J^ABS^/RC means that more light is absorbed *per* Q_A_-reducing RCII in Ws-4 than in Col-0. Taking into account that the flux of dissipated energy (J^DI^/RC) was not significantly different in the three accessions, the additional photons absorbed by Ws-4 antenna were used within the RCII to inject more electrons into the linear electron transport chain, as indicated by the significantly higher values (+5%) of J^TR^/RC and J^ET^/RC (Figure 3B). The number of electrons injected into each electron transport chain (factor N) was indeed found significantly higher (5%) in Ws-4 when compared to Col-0. In L*er*-0 slightly, but not significantly, lower values were found for every flux compared to Col-0. These data indicate a slightly higher maximum activity of RCII in dark-adapted Ws-4 plants, resulting in a more efficient closure by saturation pulses.

**Figure 4:**
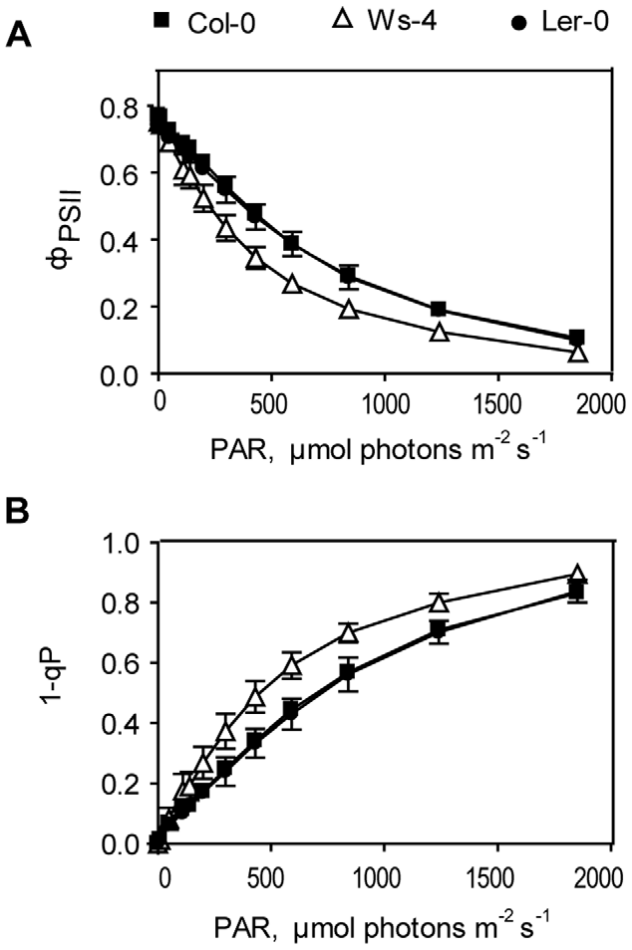
Rapid light response curves of PSII quantum yield and excitation pressure in Col-0, Ws-4 and L*er*-0 accessions. Leaves from plants grown hydroponically at an irradiance of 120 µmol photons m^−2^ s^−1^ were detached and illuminated stepwise using photosynthetically active radiation (PAR) of various intensities. Chlorophyll fluorescence was measured, and the quantum yield of PSII photochemistry **Φ**_PSII_ (A) the excitation pressure 1-qP (B) were calculated. The data are plotted *versus* irradiance and represent means ±SD (n = 7). SD bars are shown when larger than the symbols.

To investigate the actual (effective) performance of PSII in the three accessions, rapid response light curves of the quantum yield of PSII photochemistry (Φ_PSII_) were recorded in plants adapted to GL conditions. This parameter, which is an indicator of the efficiency at which light absorbed by PSII is used for photochemistry in a light-adapted plant, had initially similar values in the three accessions (approx. 0.75). Φ_PSII_ decreased while increasing the intensity of the photosynthetically active radiation (PAR), but significantly lower values were obtained for Ws-4 (Figure 4A). The parameter 1-qP, also known as PSII excitation pressure, is an estimate of the proportion of closed PSII centers, which reflects the redox state of the photosynthetic electron transport chain [21]. Consequently, this parameter should be sensitive to PAR intensity. The three accessions initially displayed 1-qP values of approx. 0.015 (Figure 4B). PSII excitation pressure exhibited a light saturation response to increasing PAR intensities, and resulted in an apparent quantum requirement of about 450 µmol photons m^−2^ s^−1^ absorbed to close 50% of the PSII centers in Ws-4 as compared to about 700 µmol photons m^−2^ s^−1^ in the other two accessions. These results indicate a lower effective performance of PSII electron transport in continuous light due to an increased efficiency of PSII closure for Ws-4. This information together with the higher maximum activity of RCII obtained in Ws-4 from the OJIP data when applying saturation pulses, provide the first indications of an increased sensitivity of PSII to HL stress in this accession.

### Photosynthetic pigment composition and PSII photoprotection

**Figure 5:**
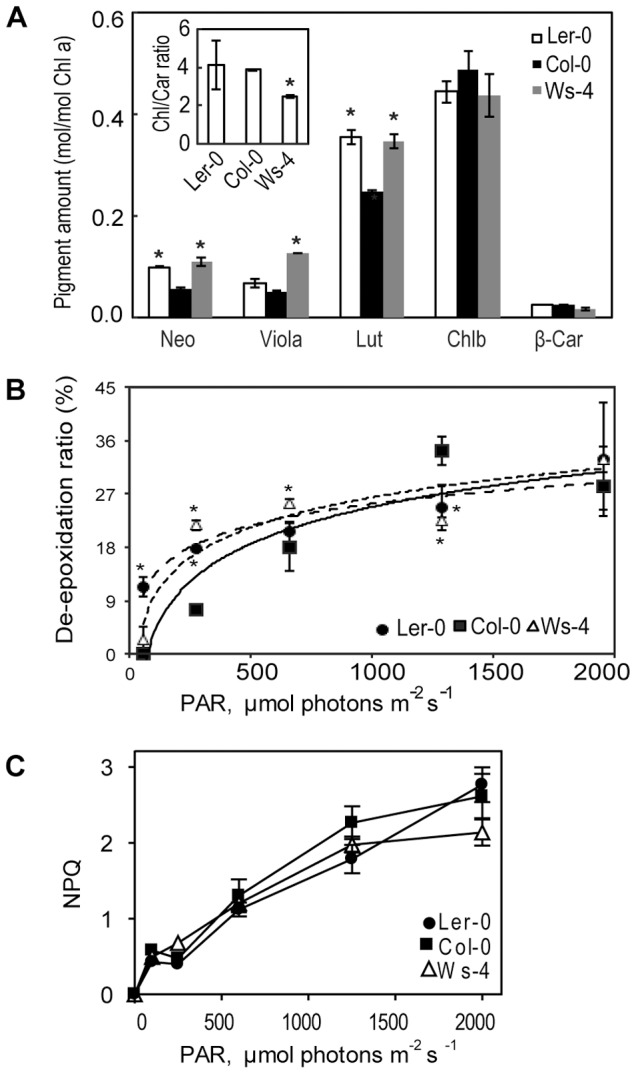
Photosynthetic pigment composition and photoprotection in Col-0, Ws-4 and L*er*-0 accessions. (A) Leaves from plants grown on soil at an irradiance of 120 µmol photons m^−2^ s^−1^ were detached at the end of the dark period, and the pigment composition was determined by HPLC. Amounts of main pigments are expressed relative to Chl *a.* Neo, neoxanthin; Viola, violaxanthin; Lut, lutein; Chl *b*, chlorophyll *b*; ß-car, ß-carotene. The insert presents the total Chl/total Carotenoid ratio. (B) Plot of de-epoxidation ratio (DR) *versus* irradiance. For determination of xanthophyll cycle activity, leaves were illuminated for 20 min at defined PAR intensity (for 10 min at PAR>1000 µmol photons m^−2^ s^−1^), then the pigments were extracted and quantified. De-epoxidation ratio was calculated as (antheraxanthin+zeaxanthin)/(antheraxanthin+zeaxanthin+violaxanthin) and expressed in %. The data plotted in A and B are means ± SD (n = 3–5). The significant differences are indicated by asterisk (Student's t-test, P<0.05). (C) Plot of *NPQ versus* irradiance. Chlorophyll fluorescence was measured in leaves illuminated for 20 min at various PAR intensities. *NPQ* was calculated as (*F*_m_ *−F*_m_ *′*)/*F*_m_ *′* and plotted as means ±SD (n = 5–7). SD bars are shown when larger than the symbols.

The qualitative photosynthetic pigment (Chl and carotenoid) composition in leaves from GL plants was found identical in the three accessions (Figure 5A), and typical for healthy green leaves [22]. The most abundant pigments were Chl *a*, Chl *b* and lutein. Lutein-5,6-epoxide and Chl epimers were only present in traces, and were therefore not quantified. The total amount of antheraxanthin together with zeaxanthin was found larger in L*er*-0 than in Ws-4 and Col-0, but still much lower than the amount of β-carotene (Figure S3). When expressed on Chl *a* basis, the amounts of xanthophylls, *i.e.,* neoxanthin, violaxanthin, lutein and zeaxanthin, differed among the accessions. Neoxanthin and lutein were found 1.5–2 fold more abundant in Ws-4 and L*er*-0 as compared to Col-0 (Figure 5A). Ws-4 distinguished itself in the relative violaxanthin content, which was about two-fold higher than in the other two accessions. This explains the different Chl-to-carotenoid molar ratio in Ws-4 (approx. 2.5) as compared to Col-0 and L*er*-0 (approx. 4) (insert Figure 5A), resulting in a 40% decrease in Ws-4.

Next experiments were designed to study the xanthophyll cycle activity and thermal photoprotection in leaves illuminated for 20 min at various PAR intensities. The de-epoxidation ratio (DR) at PAR of 53 µmol photons m^−2^ s^−1^ was found higher in L*er*-0 (Figure 5B). At intermediate PAR intensities up to about 1,000 µmol photons m^−2^ s^−1^, both L*er*-0 and Ws-4 displayed slightly but significantly higher DR than Col-0. At 2,000 µmol photons m^−2^ s^−1^ there were, however, no large differences in DR among the three accessions. Chl fluorescence was also recorded in leaves illuminated for 20 min at various PAR intensities, and the non-photochemical quenching parameter (*NPQ*) was calculated. Light curves of *NPQ* revealed similar levels of thermal photoprotection (Figure 5C) despite quantitative variation in xanthophyll pigments. Since the violaxanthin-to-Chl molar ratio was two-fold higher in Ws-4, the additional violaxanthin pool could have another role than photoprotection in the thylakoid membrane, such as the control of membrane organization and modulation of LHCII antenna efficiency (for reviews, see refs. [1], [14], [23]–[25]).

### Levels of photosynthetic proteins and PSII phosphoproteins under GL conditions

**Figure 6:**
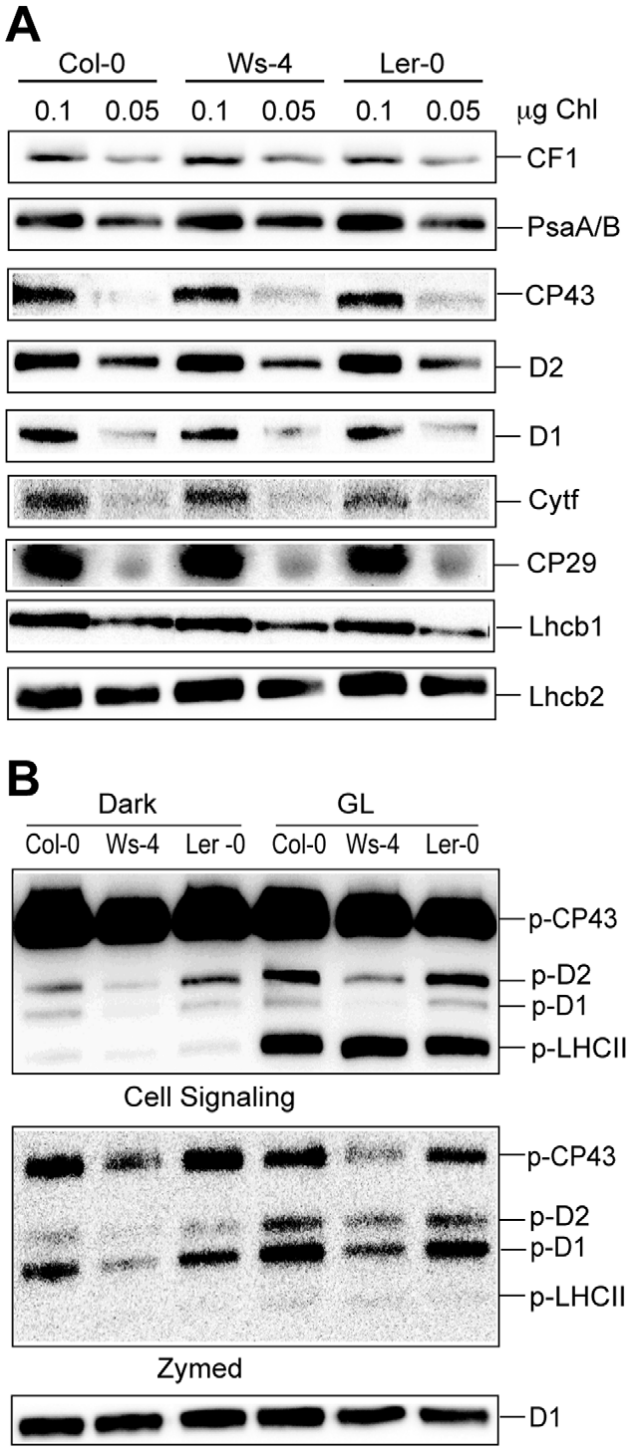
Levels of thylakoid photosynthetic proteins in Col-0, Ws-4 and L*er*-0 accessions. Thylakoid membranes were isolated from plants grown hydroponically at an irradiance of 120 µmol photons m^−2^ s^−1^ (GL). (A) Representative western blots with antibodies against various photosynthetic proteins. The loaded amount of Chl (µg) is indicated above each lane. (B) Phosphorylation of PSII proteins was assessed in thylakoid membranes isolated in the presence of 10 mM NaF from plants dark-adapted for 16 h or exposed for 3 h to GL. Representative western blots with anti-phosphothreonine antibody from Cell Signaling and Zymed and with anti-D1 antibody (as loading control) are shown. The gels were loaded with 0.25 µg of Chl in each well. The positions of the major phosphorylated PSII proteins are indicated.

Next we investigated the levels of the four major photosynthetic complexes in the thylakoid membrane. Western blotting of thylakoid membrane proteins separated in SDS-gels loaded on equal Chl basis indicated no apparent difference among the studied accessions in the levels of the CF1 β-subunit of the ATP synthase, the PsaB subunit of PSI complex, the CP43, D2, D1, CP29, Lhcb1 and Lhcb2 subunits of PSII complex, and the Cytf subunit of the cytochrome b_6_f complex (Figure 6A).

To test whether the steady-state phosphorylation levels of PSII proteins were affected in the three accessions, western blotting was performed for thylakoids isolated from plants, which were either dark-adapted for 16 h or following illumination for 3 h with GL, using anti-phosphothreonine antibodies from two different manufacturers, namely Cell Signaling and Zymed (Figure 6B). Only weak phosphorylation was observed in darkness for the LHCII proteins. Under GL conditions, these proteins were mainly phosphorylated, whereas the PSII core D1/D2 proteins were phosphorylated to a lower extent. The PSII core CP43 protein was found phosphorylated in both darkness and GL conditions. Control western blots with anti-D1 indicated equal loading (Figure 6B). If considering the level of phosphorylated PSII core proteins in Col-0 as 100%, the levels of immunodetected phospho-D1/D2 proteins were about 50% lower in Ws-4, whereas the level of phospho-CP43 was found less affected (about 25%). L*er*-0 displayed similar levels of phosphorylated PSII core proteins to Col-0. No difference in the levels of phospho-LHCII proteins was observed among the accessions. Despite distinct affinities for the PSII proteins, similar pattern of phosphorylation was obtained with the two different anti-phosphothreonine antibodies (Figure 6B).

### Levels of PSII phosphoproteins and of the involved kinases under HL conditions

**Figure 7:**
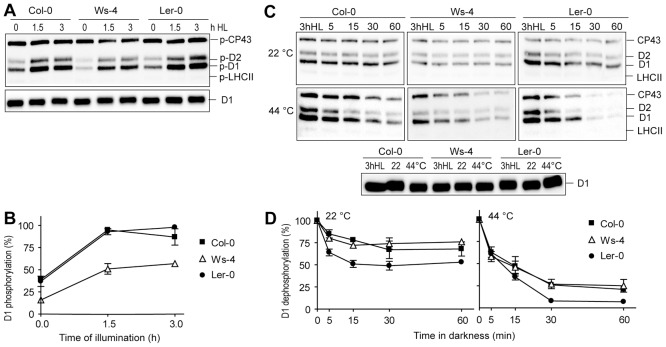
Time course of PSII protein phosphorylation and dephosphorylation in Col-0, Ws-4 and L*er*-0 accessions. (A) *In vivo* steady-state PSII protein phosphorylation. Intact plants grown hydroponically were illuminated for 0, 1.5 and 3 h with high light (HL = 950 µmol photons m^−2^ s^−1^), followed by isolation of thylakoids in the presence of NaF, and western blot analysis with Zymed antibody. A western blot of the same samples was performed with anti-D1 antibody, and used as a loading control. (B) Plot of data shown in panel A, where quantification levels are expressed relative to phosphorylation in Col-0. (C) *In vitro* PSII protein dephosphorylation. Thylakoid membranes were isolated from 3 h HL treated plants in the presence of NaF, and incubated in darkness at 22° or 44°C for the indicated periods of time. Samples were blotted using a Zymed antibody. The western blot with anti-D1 antibody of samples from 3 h HL plants and incubated in darkness at 22° or 44°C for 60 min indicates equal loading of the gels. (D) Plot of C, representing % remaining D1 phosphorylation, 100% = initial phosphorylation level in each accession. The plotted data in (B) and (D) are means ±SD (n = 3). SD bars are shown when larger than the symbols.

Reversible protein phosphorylation is an important photoprotective mechanism in the thylakoid membrane upon changes in light quality and intensity. The STN7 protein kinase is involved in phosphorylation of LHCII, CP29, CP26 and TSP9 proteins under low light conditions, whereas the STN8 kinase phosphorylates PSII core D1, D2, CP43 and PsbH proteins under HL conditions (for reviews, see refs. [26], [27]). To further study the interesting difference in the phosphorylation of the D1/D2 proteins, western blotting was performed for thylakoids isolated from plants illuminated for 1 h and 3 h with HL (Figure 7A). Control western blots with anti-D1 antibody indicated equal loading. Considering maximum level of D1 phosphorylation in Col-0 as 100%, about 50% less phosphorylation was immunodetected in HL-treated Ws-4 plants as compared to the other two accessions (Figure 7B).

Next the phosphothreonine blot results were verified using quantitative mass spectrometry. For this purpose, thylakoids isolated from plants exposed for 3 h to HL were treated with trypsin to cleave the surface-exposed phosphorylated and non-phosphorylated parts of the membrane proteins, which were then separated and quantified using liquid chromatography and mass spectrometry. To obtain accurate quantitative data the normalization procedure accounting for the differences in signal intensities of phosphorylated and corresponding non-phosphorylated peptides was used [28]. The ratios of normalized signals for each phosphopeptide/peptide pair determined the extent of phosphorylation for the D1, D2, CP43 and PsbH proteins of PSII in thylakoids from each accession. Phosphorylated D1/D2 decreased by 50% in Ws-4 as compared to Col-0, while phospho-CP43, as well as singly and doubly phosphorylated PsbH were found at similar levels in all three accessions (Table 3).

**Table 3:** Quantitative LC-MS of PSII core protein phosphorylation of Col-0, Ws-4 and L*er*-0 accessions.

Protein	Col-0	Ws-4	L*er*-0
P-CP43	100%	100±7%	100±14%
P-D1	100%	50±12%*	100±15%
P-D2	100%	48±8%*	98±8%
P-PsbH	100%	95±11%	102±9%
PP-PsbH	100%	96±12%	100±16%

Data represent means ±SD of four independent preparations.

* , Significantly different from Col-0 and L*er*-0 (Student's t-test P<0.05).

The plants were grown hydroponically for six weeks at an irradiance of 120 µmol photons m^−2^ s^−1^ and treated for 3 h at an irradiance of 950 µmol photons m^−2^ s^−1^. Thylakoid membranes were isolated in the presence of NaF. The levels of phosphorylated PSII core proteins were analyzed by quantitative mass spectrometry and expressed relative to Col-0.

To study the activity of the PSII core protein phosphatase, *in vitro* dephosphorylation experiments were carried out at 22°C and 44°C, the latter chosen since it is known that the phosphatase is activated by such elevated temperature [29]. Western blots with anti-phosphothreonine antibodies of thylakoids at both temperatures indicated that D1 protein dephosphorylation proceeds with similar time course in Ws-4 and Col-0, and is faster in L*er*-0 (Figure 7C and D). It is not clear at present whether the faster dephosphorylation observed in L*er*-0 has any physiological relevance since the steady state levels of PSII phosphoproteins were found similar in L*er*-0 and Col-0 (Figure 7A). These results indicate that the reduced D1/D2 phosphorylation level in Ws-4 is not due to dephosphorylation reactions.

**Figure 8:**
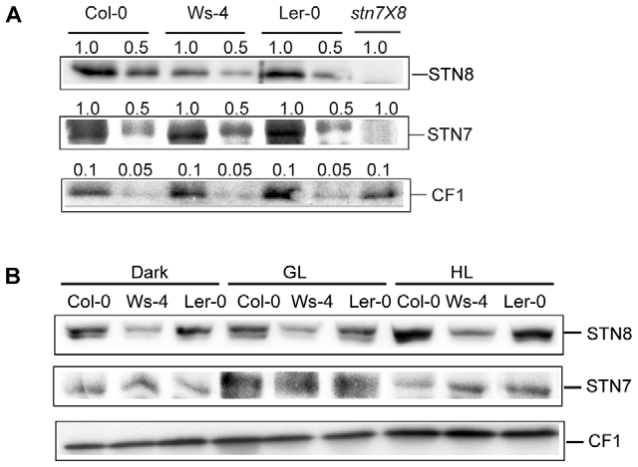
Levels of thylakoid protein STN8 and STN7 kinases in Col-0, Ws-4 and L*er*-0 accessions. (A) Thylakoid membranes were isolated from plants grown hydroponically at an irradiance of 120 µmol photons m^−2^ s^−1^ (GL), and analyzed by western blotting with STN8 and STN7-specific antibodies. Last lane is loaded with thylakoids isolated from an *stn7xstn8* mutant for validating the specificity of the antibodies used. The loaded amount of Chl (µg) is indicated above each lane. (B), Representative western blots with STN8 and STN7 antibodies of thylakoids (1 µg Chl/lane) isolated from plant which were 16-h dark-adapted, illuminated for 3 h with GL or with high light (HL = 950 µmol photons m^−2^ s^−1^). Western blots with anti-CF1 antibody are shown in both panels as loading controls.

The observed difference in the steady state phosphorylation levels could be due to a difference in the relative levels of involved kinases. Western blotting of SDS-gels loaded on Chl basis with an STN7-specific antibody revealed similar levels of this kinase (Figure 8A). In similar western blots developed with an STN8-specific antibody, the corresponding kinase was found in amounts reduced by about 50% in Ws-4 as compared to Col-0 and L*er*-0. No cross-reaction with either of these antibodies was obtained in thylakoids isolated from an *Arabidopsis* mutant lacking both STN7 and STN8 kinases (*stn7xstn8*, [4]), validating the specificity of these antibodies. Control western blots with anti-CF1 antibody indicated equal loading.

Next the immunodetected STN8 kinase levels in thylakoids isolated from 16-h dark-adapted plants were compared to those from plants exposed for 3 h to GL or HL conditions (Figure 8B). In all three accessions, the amount of STN8 was found about 1.5-fold higher in HL than in either dark or GL conditions. Nevertheless, the amount of STN8 kinase in Ws-4 was lower (by about 50%) in all tested conditions as compared to Col-0 and L*er*-0, thus resembling the pattern observed for PSII core protein phosphorylation (Figure 7A). For comparison, the level of STN7 was found higher in GL than in the dark or HL conditions, and to a similar extent in all three accessions (Figure 8A and B). Control western blots with anti-CF1 antibody indicated equal loading. Taken together, these results indicate that the increase in STN8 abundance is a photoacclimation strategy, but yielding lower STN8 levels in Ws-4.

### PSII function and plant growth under HL conditions

**Figure 9:**
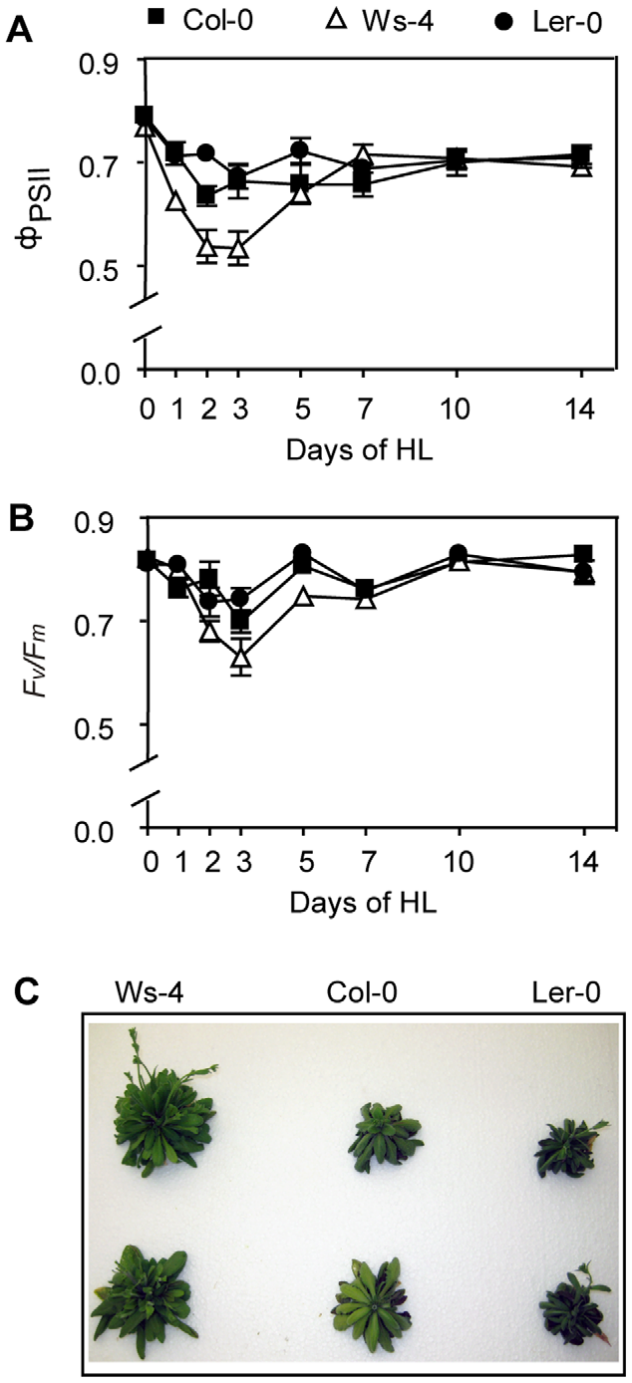
PSII activity of Col-0, Ws-4 and L*er*-0 accessions during growth under high-light conditions. The plants were initially grown hydroponically for four weeks at 120 µmol photons m^−2^ s^−1^, and then transferred to 950 µmol photons m^−2^ s^−1^ (HL) for additional two weeks. PSII activity was measured *via* chlorophyll fluorescence. The Φ_PSII_ parameter in detached leaves under light (A) and the *F*_v_/*F*_m_ parameter following 15 min of dark-adaptation (B) are plotted as a function of number of days in HL. The data are means ±SD (n = 5). SD bars are shown when larger than the symbols. (C) Appearance of plant health during growth under HL conditions. Two plants from each accession were photographed after the additional two weeks of growth under HL conditions.

To investigate if there is a difference in the sensitivity of PSII to HL stress among the three accessions, plants were first grown for four weeks under GL conditions, and then transferred to HL conditions for two weeks. As shown in Figure 9A, Ws-4 plants displayed about 20% lower quantum yield of PSII photochemistry (Φ_PSII_) in the first 3 days of HL growth when compared to Col-0 and L*er*-0. During the remaining HL period, Ws-4 has gradually recovered its PSII activity reaching similar levels to Col-0 and L*er*-0. A similar pattern was observed for the maximum quantum yield of PSII activity (*F*_v_/*F*_m_), although the decrease was only by 10% (Figure 9B), indicating a slightly increased sensitivity to HL of PSII in Ws-4. Figure 9C presents photographs of the plants at the end of the HL period. Ws-4 had significantly larger shoot weight (4.95±0.26 g) than Col-0 (3.41±0.43 g), and L*er*-0 (2.42±0.18 g) (n = 5, Student's t-test, P<0.05). These values are about 60% lower than the ones obtained for the shoot weight of six weeks old plants grown hydroponically under GL conditions (Table 1).

### PSII dynamics and chloroplast ultrastructure under HL conditions

**Figure 10:**
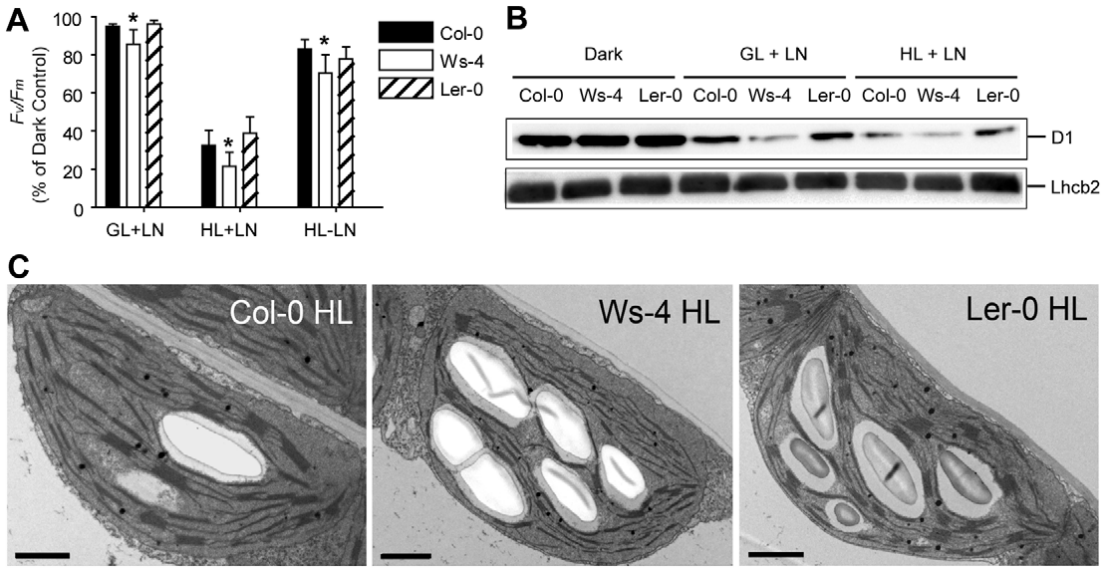
Photoinhibition of PSII in Col-0, Ws-4 and L*er*-0 accessions. Leaves detached from plants grown hydroponically at an irradiance of 120 µmol photons m^−2^ s^−1^ (GL) were exposed for 3 h to GL or high light (HL = 950 µmol photons m^−2^ s^−1^) treatments in the presence or absence of lincomycin (LN). (A) The *F*_v_ */F*_m_ parameter was determined in leaves after 5 min dark adaptation. The levels of *F*_v_ */F*_m_ were calculated relative to a value of 0.82, obtained in 16 h dark-adapted Col-0 leaves, and plotted as means ±SD (n = 3). (B) Thylakoid membranes were isolated from the treated leaves and subjected to western blotting with anti-D1 and with anti-Lhcb2 (as loading control) (0.25 µg Chl/lane). (C) Transmission electron micrographs of chloroplast ultrastructure from plants exposed to 3 h HL. Scale bar: 1 µm.

The increased sensitivity of PSII in Ws-4 during growth under HL conditions prompted us to investigate the steps in PSII repair cycle preceding *de novo* D1 protein synthesis. For this purpose, we have used GL- and HL-treated leaves in the presence of lincomycin, an inhibitor of D1 protein synthesis. PSII activity, measured *via* the *F*_v_ */F*_m_ parameter, decreased only slightly, *i.e*., by 5% in Col-0 and L*er*-0 and by 15% in Ws-4 leaves illuminated with GL (Figure 10A). The decrease in the *F*_v_ */F*_m_ parameter was more pronounced for leaves illuminated with HL, *i.e.,* by 70% for Col-0 and L*er*-0 and by 80% for Ws-4. Although small, the difference in 10% between Ws-4 and the other accessions was found significant. In leaves treated with HL in the absence of lincomycin, the PSII centers were still found more inactivated in Ws-4 (by 30%) as compared to Col-0 and L*er*-0 (20%). Enhanced D1 protein degradation was immunodetected in Ws-4 as compared to Col-0 and L*er*-0 in both GL and HL in the presence of lincomycin (Figure 10B). Control western blots with anti-Lhcb2 antibody indicated equal loading. Taken together, these results provide evidence that PSII in Ws-4 is slightly but significantly more susceptible to photoinhibition. The reason for this is the enhanced inactivation/degradation, which exceeds the capacity for *de novo* D1 protein synthesis to a higher extent in Ws-4 than in the other two accessions.

Leaves from plants grown under GL and subsequently exposed for 3 h to HL have been also studied in terms of chloroplast ultrastructure using electron microscopy (Figure 10C). Notably, Ws-4 displayed reduced height and width (diameter) of the grana as compared to the other two accessions (*i.e.,* approx. 5 *versus* 6 appressed thylakoid lamellae *per* granum on average, Table S2). When compared with the electron microscopy data obtained at GL conditions (Figure 1C; Table S2), it is obvious that HL treatment has reduced the number of grana/plastid and the grana size (length and/or width) in all three accessions, but this process appeared significantly more pronounced in Ws-4.

**Figure 11:**
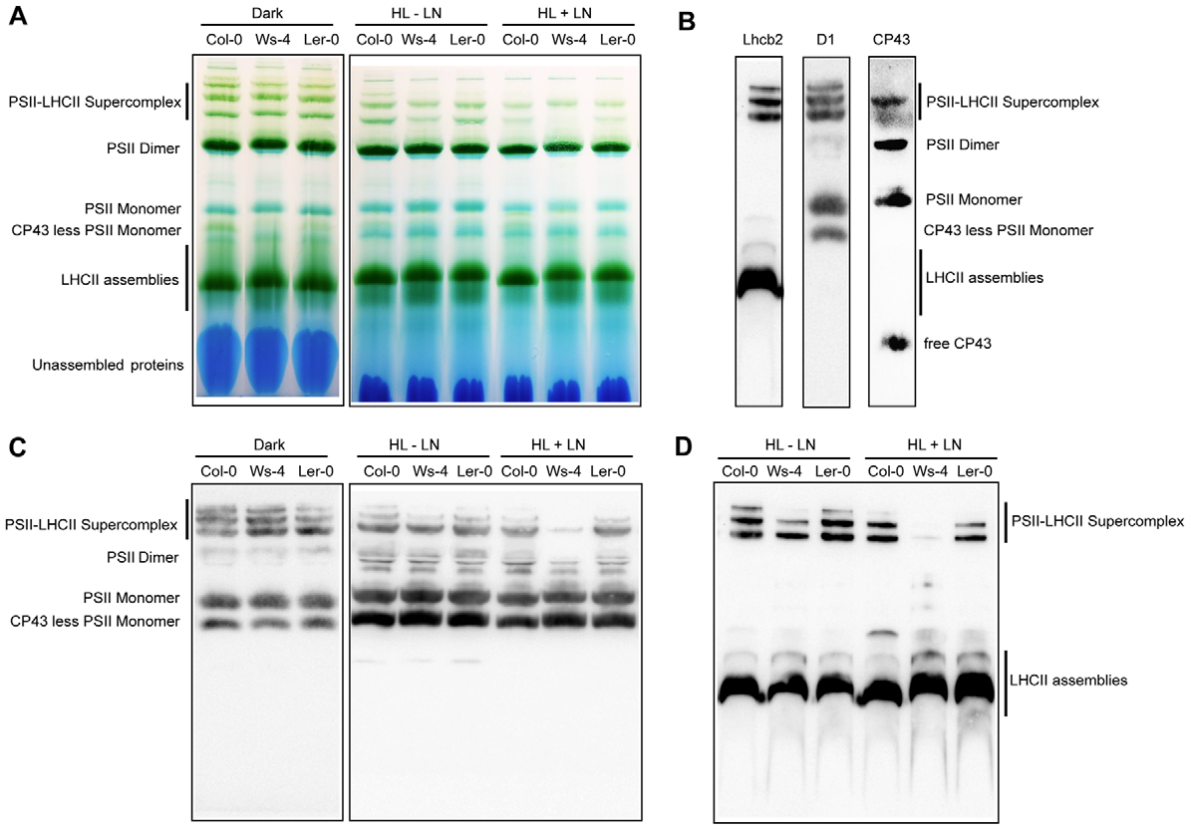
Analysis by Blue-native gel electrophoresis of thylakoid protein complexes from Col-0, Ws-4 and L*er*-0 accessions. Leaves detached from plants grown hydroponically at an irradiance of 120 µmol photons m^−2^ s^−1^ (GL) were exposed to high light (HL = 950 µmol photons m^−2^ s^−1^) in the absence or presence of lincomycin (LN) for 3 h. As control, 16 h dark-adapted plants were used. Thylakoid membranes were isolated and solubilized mildly with n-dodecyl-ß-D-maltoside, and the various types of Chl protein complexes were separated by Blue-native gel electrophoresis. (A) Representative unstained Blue-native gel (8 µg Chl per lane). (B) (C). The PSII complexes together with various combinations of LHCs were identified based on western bloting with anti-Lhcb2, D1 and CP43 antibodies, and based on previous reports [2], [33]. Representative western blot with anti-D1 antibody of gel as in (A). (D) Representative western blot with anti-Lchb2 of gel as as in (A).

Blue-native polyacrylamide gel electrophoresis (BN-PAGE) has been widely used to separate under non-denaturing conditions individual membrane protein complexes of isolated thylakoids solubilized with mild detergents, such as n-dodecyl-ß-D-maltoside and digitonin. Using this method, a high diversity of PSII complexes has been found in the thylakoid membrane, and it has been shown that dodecylmaltoside is more efficient than digitonin to extract the PSII complexes [30], [31]. Using western blotting and/or mass spectrometry, four types of PSII complexes could be identified, with various distribution in the thylakoid membrane, namely PSII-LHCII supercomplexes, PSII core dimers, PSII core monomers, and CP43-less PSII core monomers [2], [32], [33]. PSII supercomplexes are the largest, contain LHCII in various combinations and are found in the grana regions. PSII core dimers also found in the grana, but lack LHCII and migrate close to PSI in BN-gels. PSII core monomers contain assembled CP43 and predominate in stroma thylakoids. CP43-less core monomers are the smallest PSII complexes and are exclusively found in the stroma thylakoids. In this study, the four types of PSII complexes together with various combinations of LHCs, were separated by BN-PAGE from dodecylmaltoside solubilized thylakoids from plants which were 16 h dark-adapted or following exposure for 3 h HL in the presence or absence of lincomycin (Figure 11A). The identity of PSII complexes was established based on western blotting with antibodies against a major light harvesting protein Lhcb2 protein, the PSII RC D1 protein and the PSII core CP43 subunit (Figure 11B), and based on previous reports [2], [33]. The unstained BN-gel shows that the levels of two types of PSII complexes changed upon transfer from darkness to 3 h HL, as follows: the bands containing PSII supercomplexes decreased in intensity, whereas the CP43-less complex band increased in intensity. No large differences in the distribution of various PSII complexes among the three accessions were observed in BN gels (Figure 11A). Nevertheless, the corresponding western blots with anti-D1 antibody clearly show that specifically under HL conditions in the presence of lincomycin, the PSII supercomplex form was less abundant in Ws-4 than in Col-0 and L*er*-0 (Figure 11C). The Lhcb2 western blot of PSII also revealed reduced amounts of this protein in the supercomplexes after the high light treatment in the presence of lincomycin (Figure 11D).

The relative amounts of the four PSII complexes in each lane loaded with solubilized thylakoids from each treated sample were estimated by quantification of D1 western blots, and are presented in Table S4. In the presence of lincomycin 3 h HL-treated Ws-4 plants contained 5% of the total D1 protein in PSII dimers and supercomplexes as compared to 20% and 12% in Col-0 and Ler-0, respectively. Instead, the CP43-less monomers were found slightly but significantly more abundant in Ws-4 (55% as compared to 45% and 46%, respectively). The calculated ratio of PSII monomer to dimer was approx. 20∶1 in Ws-4 as compared to 4∶1 and 7∶1 in Col-0 and L*er*-0. These data indicate the highest extent of PSII disassembly during HL stress in Ws-4 as compared to the other two accessions, which is in line with the finding of enhanced PSII inactivation/D1 degradation (Figure 10) in this accession.

## Discussion

Col-0, Ws-4 and L*er*-0 are three *Arabidopsis* accessions, which are widely studied in comparison with insertional mutants in the same background. Only a few reports are available about the individual photosynthetic performance of *Arabidopsis* accessions in relation to each other [10], [11], and Ws-4 was thus far not studied at all. In this study, various biophysical, biochemical, ultrastructural and physiological methods were employed to characterize PSII performance in the three accessions under GL and HL conditions. The major findings are that PSII complexes in Ws-4 are slightly but significantly more susceptible to HL, and that the phosphorylation of D1/D2 proteins displays reduced steady state levels in this accession. The primary reasons for the observed differences are the increased lipid-to-Chl and carotenoid-to-Chl ratios and the reduced amount of STN8 kinase in the thylakoid membrane, respectively.

### Impact of thylakoid lipid-Chl-carotenoid stoichiometry on PSII function

Immunoblot analyses of gels loaded on Chl basis showed no difference in the relative amounts of photosynthetic complexes in the three accessions (Figure 6). The similar values for PSI to PSII ratio (Table 1) are also in line with this conclusion. This information together with the approx. 30% higher thylakoid lipid-to-Chl ratio in Ws-4 (Figure 2) indicate that the Chl-protein complexes are more ‘diluted’ with MGDG and DGDG in the leaves of this accession. In contrast with DGDG, a bilayer-forming lipid, MGDG is a non-bilayer lipid, since it is conically shaped, and exerts a lateral pressure within the hydrophobic space of the membrane [16]. A similar increase in the proportion of MGDG and DGDG was obtained in Ws-4 leaves (30–40%) as compared to the other two accessions (Figure 2A). This indicates that the additional MGDG does not form a separate non-bilayer H_II_ (hexagonal inverted micelles) phase, but is ‘forced’ to stay in the bilayer in Ws-4 thylakoids. Free xanthophylls (neoxanthin, violaxanthin, lutein and zeaxanthin) have been proposed to stabilize bilayer formation of MGDG by lowering the lateral pressure, thus triggering a more diluted and random distribution of PSII supercomplexes in the grana regions of the thylakoid membrane [14]. Therefore, we suggest that in the thylakoid membrane of Ws-4, containing more MGDG and DGDG, the protein complexes are less dense but more randomly distributed. This could explain the more efficient closure of PSII RCs upon application of either saturation pulses or continuous light (Table 2; Figure 4). This may be the ‘price’ Ws-4 must pay to allow easier lateral migration of damaged complexes to the sites of repair (see Discussion, next section).

Xanthophylls are intrinsic components of the LHCII antenna and are mainly involved in regulation of light harvesting and photoprotection, but also in the control of membrane organization (for reviews, see refs. [1], [14], [23]–[25]). In addition to the presence of more lipids *per* Chl, we also determined a Ws-4-specific difference in the violaxanthin content relative to Chl, leading to a 40% lower Chl/carotenoid ratio as compared to the other two accessions (Figure 5). Based on their structure, xanthophylls are considered amphiphilic molecules similar to membrane lipids. Violaxanthin (and neoxanthin) are more polar than lutein and zexanthin. From the analysis of xanthophyll biosynthesis mutants, it has been shown that the PSII quantum efficiency increases with the polarity of the xanthophyll bound to LHCII, since the lifetime of the Chl excited state is prolonged (for a review, see ref. [24]). The details of this alteration regulating the efficiency of light harvesting have not been yet clarified. At present stage, we cannot discern between bound and free pool of additional violaxanthin in Ws-4. Nevertheless, the slightly but significantly higher maximum PSII activity in Ws-4, as indicated by the J^ABS^, J^TR^ and J^ET^ fluxes *per* RC (Figure 3), indicates that at least a small fraction of the additional violaxanthin may be bound to LHCII-PSII supercomplexes and participate in RCII excitation. The largest fraction of additional violaxanthin may be free and used to stabilize the MGDG-enriched membrane. In this case, the dilution of PSII complexes with additional lipids in the membrane is sufficient to trigger the faster closure of RCII centers (Table 2; Figure 4) and enhance the photoinhibitory damage (Figures 9 and 10) in Ws-4.

Zeaxanthin was shown to be of primary importance for photoprotection [34], [35]. The zeaxanthin content and DRs were higher in Ws-4 but also in L*er*-0 at low PAR intensities (Figure 5). At high PAR intensities no difference in these parameters were observed among the accessions, and there was no difference in *NPQ* for the entire studied PAR intensity range. Although higher violaxanthin-to-Chl ratio was obtained in Ws-4, the additional violaxanthin appeared not to act as a substrate for violaxanthin de-epoxidase. This indicates that *NPQ*-related photoprotection is not different in Ws-4, and that xanthophyll cycle may be only one component in the formation of *NPQ* [25]. Taken together, the distinct lipid-Chl-carotenoid ratio is the most likely reason for the enhanced photoinhibitory damage to PSII complexes in Ws-4.

### Impact of STN8-Chl-lipid stoichiometry on PSII dynamics

The PSII core D1/D2 proteins were found 50% phosphorylated in Ws-4 relative to Col-0 and L*er*-0, whereas phosphorylation of CP43 was marginally affected and that of LHCII not affected at all in the three examined conditions: 16 h dark, 3 h GL and 3 h HL (Figures 6 and 7). Based on the western blot data presented in Figure 7A, we suggest that the major reason for the inability of Ws-4 to fully phosphorylate the D1/D2 proteins is the 50% reduction in the abundance of the STN8 kinase relative to Chl (Figure 8). The phosphorylation level of CP43 was less affected by the reduction in the STN8 kinase level, supporting recent indications for the involvement of both STN7 and STN8 kinases [26].

At steady state conditions, under different light regimes, complex regulatory mechanisms become active and serve to yield optimal overall rates of photosynthesis. In this context, the relative abundance of the STN8 protein in the thylakoid membrane, as judged from our western blots, increased in all three accessions upon transfer from dark to HL, but not upon transfer to GL conditions. The relative level of the STN7 protein increased in all three accessions upon transfer from dark to GL, but not upon transfer to HL conditions. In other words, the STN8 and STN7 kinases are more abundant under conditions where they are active. The control of their amount is probably a component of the long-term acclimation response. In the case of STN7 or its *Chlamydomonas* homologue Stt7, it has been suggested that their amounts are regulated by the redox status of the electron transport chain and by phosphorylation [36], [37]. Upon transfer from dark to HL conditions, Ws-4 increased the relative amount of STN8 but not to Col-0 levels (Figure 8). The factors regulating the amount of STN8 kinase in the membrane and those responsible for the observed reduction in Ws-4 deserve further investigation.

The relevant question in this study is about the consequences of a reduced STN8 content in Ws-4 for the steps in the PSII repair cycle, which depend on PSII core protein phosphorylation, namely disassembly of the complex, D1 degradation and thylakoid unstacking. It has been proposed that the phosphorylation of PSII core proteins by STN8 plays a major role in controlling the lateral movement of PSII-LHCII supercomplex along the thylakoid membrane to the sites of repair [3], [27]. This conclusion was supported by the fact that *Arabidopsis* mutants lacking the STN8 protein (*stn8*) display only residual D1/D2 protein phosphorylation, accumulate damaged supercomplexes, and cannot degrade the D1 protein. Based on these facts, if STN8-dependent phosphorylation would be the solely factor involved in the lateral movement of the supercomplex, then an intermediate situation between *stn8* mutant and wild type plants would be expected in plants displaying intermediate phosphorylation levels such as Ws-4. However, based on the results of the present study this does not appear to be the case. HL-treated Ws-4 displayed enhanced D1 degradation and reduced relative amounts of PSII-LHCII supercomplexes as compared to the other two accessions (Figures 10 and 11). These observations cannot be explained solely by the reduced STN8 content, but by the dilution of protein complexes with additional lipids in Ws-4. Experiments manipulating Chl-to-lipid ratio have demonstrated that addition of lipids causes an increase in the diffusion of protein complexes in the thylakoid membrane [38]. Thus, the specific effects of reduced D1/D2 phosphorylation levels on PSII repair require investigations in *Arabidopsis* mutants with similar lipid-to-Chl stoichiometry and with reduced STN8 kinase content.

Dynamics in stacking of the thylakoid membrane is a mechanism to facilitate lateral membrane protein diffusion and PSII repair [4]–[6]. In contrast with the observations of larger grana stacks in the *stn8* mutants than the wild type plants, there were no large differences in the thylakoid membrane stacking of GL-type of plants, and only slightly smaller grana stacks were observed in HL-treated Ws-4 plants than in the other two accessions (Figures 1 and 10; Table S2). Therefore, the change in thylakoid organization appears to be an ON/OFF mechanism in relation to PSII protein phosphorylation, meaning that even partial phosphorylation may be sufficient to preserve the normal ultrastructure. Whether the addition of lipids *per* Chl represents a mechanism for thylakoid membrane stacking compensating for the full complement of STN8 deserves further investigation.

It has been proposed that protein phosphorylation switches the membrane system to a more fluid state, allowing PSII repair to occur. This was based on the observation that photoinhibition-induced mobility of complexes from grana to stroma regions of the thylakoid membrane is considerably restricted in *stn8* mutants leading to accumulation of damaged PSII supercomplexes [39]. In our study, HL-treated Ws-4 plants contain only small amounts of supercomplexes, and accumulate instead largest proportion of CP43-less monomers, the subtype of PSII complex undergoing D1 degradation (Figure 11; Table S4). This indicates that the dilution of protein complexes by additional lipids facilitates disassembly and migration of the damaged complexes to the sites of repair, thus compensating for the inability to fully phosphorylate the PSII core proteins. Despite this compensation strategy for lateral mobility in the membrane, the additional damage to more diluted complexes exceeds the D1 protein synthesis capacity, leading to slightly enhanced photoinhibition in Ws-4.

## Conclusions

Here we have used functional and physiological approaches to characterize in detail PSII performance in three widely used *Arabidopsis* accessions. The data obtained revealed that within the set of accessions analyzed, Ws-4 displays distinct PSII performance and dynamics mainly due to more diluted Chl-protein complexes in the thylakoid membrane, making this accession more susceptible to HL stress. Ws-4 also displayed reduced STN8 protein kinase content, leading to less PSII D1/D2 phosphorylation. The presented data are valuable since they provide knowledge for future investigators on the choice of background lines for mutation and PSII characterization in *Arabidopsis*. The differences observed in this study are interesting as they show that there is indeed genetic variation in Chl, lipid and STN8 kinase content, the source of which can be elucidated by further experiments, such as testing a wider range of genotypes, or screening mapping populations. This in turn will increase our understanding concerning the HL acclimation strategies of different *Arabidopsis* accessions.

## Materials and Methods

### Plant material and growth conditions

The three *Arabidopsis* accessions are the following: Ws-4 provided by the Resource Center of INRA Versailles (http://dbsgap.versailles.inra.fr/publiclines/), Col-0 and L*er*-0 ordered at the SALK SIGnAL (http://www.signal.salk.edu/cgi-bin/tdnaexpress). The three accessions showed identical anatomy with those displayed at TAIR site (http://www.arabidopsis.org/abrc/). PCR analysis using primers available at TAIR site was performed to confirm the identity of the accessions.

Plants belonging to the three accessions were grown hydroponically at 120 µmol photons m^−2^ s^−1^ (GL) at 22°C with 8 h light/16 h dark cycles, and relative humidity 70% for six weeks [40] unless otherwise indicated. For HL stress experiments, 4-weeks old plants were grown at an irradiance of 950 µmol photons m^−2^ s^−1^ for additional two weeks. For O-J-I-P and photosynthetic pigment analysis, the plants were grown on soil under GL conditions for 15 to 17 days before use. For thylakoid lipid analysis, the plants were grown on soil under GL conditions for six weeks. Chl content was measured from plants grown either hydroponically or on soil, as indicated.

### Photosynthetic pigment analysis

Chl content of whole leaves was determined by extraction in 96% ethanol and spectrophotometry according to ref. [41].

For determination of the xanthophyll cycle activity, the leaves were illuminated for 20 min with PAR of various intensities ranging between 53 and 1952 µmol photons m^−2^ s^−1^. Because long and intense illumination triggers pigment photo-destruction, the time of illumination was reduced to 10 min for PAR intensity higher than 1000 µmol m^−2^ s^−1^. After the illumination, the leaves were detached from the plants, and immediately frozen in liquid nitrogen. The samples were ground in methanol, and the extracts clarified by centrifugation at 25,000 *g* for 10 min. Each sample was dried under nitrogen gas and stored in darkness at −20°C or immediately used for HPLC analysis. For pigment extraction, all manipulations were performed at 4°C under a green safe light as recommended by Schoefs [42]. Pigment analysis was carried out on a Beckman Gold HPLC device (Beckman Coulter, USA) consisting of a solvent-delivery system (module 126) and a photodiode-array detector (module 168) [43]. The time and wavelength resolution were 1.1±0.1 s and 3 nm, respectively. The pigment extract was injected with a Rheodyne (Cotati, CA, USA) Model 7725 sample valve equipped with a 20-µl loop. Up to 20 µl of methanolic extracts were injected. Separations were carried out with a Zorbax Original reverse8-phase column (Agilent Technologies, Interchim, France, particle size of the packing 4.65 µm; 25 cm×4.6 mm I.D.) with the method described by Darko et al. [44]. The pigments were identified on the basis of their absorption spectra and their retention times described in ref. [44]. The pigment concentrations were corrected to the wavelengths of the detection (430 and 445 nm). After each measurement, the column was re-equilibrated for 20 min with the solvent mixture used initially. All HPLC analyses were carried out at 22°C and the flow-rate was 1 ml min^−1^. All solvents were of HPLC grade and purchased from Sigma-Aldrich. The xanthophyll cycle or de-epoxidation ratio was calculated as (antheraxanthin+zeaxanthin)/(antheraxanthin+zeaxanthin+violaxanthin).

### Thylakoid lipid analysis

Lipids were extracted from leaves as described [45]. The phospho- and glycolipids were separated [46] and dissolved in methanol. These lipids were analyzed by LC-MS/MS using an Agilent 1260 LC and an Agilent 6410 triple quadrupole detector. For this purpose, the lipids were separated on an RP-MS Accucore 150×2.1 mm, 2.6 µm column (Thermo Scientific) thermostated at 50°C and detected using multiple reaction monitoring for each lipid species according to [47], [48]. Neutral loss of 179, 341 and 189 in a positive mode was used for MGDG, DGDG and PG, respectively, and precursors of 225 in negative mode was used for detecting SQDG. For PG species the fatty acid composition of some of the most abundant species were verified by product ion scanning in negative mode. One minute of isocratic elution with methanol∶acetonitrile∶water (45∶40∶15, by vol.) was followed by a linear gradient of 40% isopropanol in 5 min and 80% in 20 min and maintained for 1 min. The solvent flow was kept constant at 0.25 mL min^−1^. Both solvents were supplemented with 0.2% formic acid, 0.1% ammonia and 5 µM H_3_PO_4_. The electrospray ion source was operated at 300°C and 4500 V with a nitrogen gas flow of 11 L min^−1^ at 40 psi. Fragmentor and collision cell setting were optimized for each lipid class by direct injection of pure lipids dissolved in the mobile phase.

### Thylakoid preparation and protein phosphorylation

Isolation of thylakoid membranes was performed as described in ref. [49]. To study phosphorylation of PSII proteins, 10 mM NaF was included in all buffers used for isolation of thylakoid membrane. In experiments aimed to study the dephosphorylation of PSII proteins, thylakoids were isolated from 3 h HL plants and incubated in the absence of NaF at 22° and 44°C for up to 1 h. Chl concentration was determined spectrophotometrically in 80% acetone according to Porra et al. [50].

### Chlorophyll fluorescence

Chl fluorescence at room temperature was measured using pulsed-amplitude fluorometer models PAM-210 and Dual-PAM 100 (Walz, Effeltrich, Germany).

The fast kinetics of Chl fluorescence induction were recorded according to ref. [13] on attached leaves dark adapted for 15 min. The obtained O-J-I-P transient was analyzed according to the OJIP-test [20]. The following parameters were determined from the recorded data: the maximal fluorescence yield (*F*_P_), which is equal to *F*_m_ since the excitation intensity was high enough to close all the PSII reaction centers (RC); the minimal fluorescence yield (*F*_0_) as the fluorescence yield at 45 µs; the fluorescence yield at 300 µs (*F*_300 µs_) required for the calculation of the initial slope (*M*_0_) of the relative variable fluorescence kinetics; the fluorescence yield at 2 ms (*F*_J_) and at 30 ms (*F*_I_). Using these fluorescence parameters, the energy fluxes *per* reaction center (RC) were calculated according to equations from ref. [20] and also presented below:

Flux for absorption: J^ABS^/RC = (M_0_/V_J_)/[1−(*F*_0_/*F*_m_)]

Flux for trapping: J^TR^/RC = M_0_/V_J_

Flux for dissipation: J^DI^/RC = J^ABS^/RC−J^TR^/RC

Flux for electron transport: J^ET^/RC = (M_0_/V_J_)/(1−V_J_)

with V_J_ = (*F*_J_−*F*_0_)/(*F*_m_−*F*_0_) and M_0_ = 4 (*F*_300 µs_−*F*_0_)/(*F*_m_−*F*_0_)

The index 0 refers to the time zero, *i.e.* at the onset of the fluorescence induction.

The parameter N, which reflects the total number of electrons transferred into the electron transport chain. The electron transport chain was calculated according to ref. [20].

The maximum quantum yield of PSII photochemistry, *i.e*., of open PSII centers (*F*_v_/*F*_m_) was determined as a ratio of variable fluorescence (*F*_v_ = *F*_m_−*F*_0_) to maximal fluorescence (*F*_m_) measured from detached leaves dark adapted for 15 min. A rapid response curve of photosynthesis *versus* irradiance was measured in detached leaves from light-adapted plants as previously described [13]. Briefly, PAR was increased stepwise every 20 sec, the stationary level of fluorescence in the light *F*_t_ was determined and a saturation pulse was applied to determine the maximal fluorescence in the light *F*_m_′. The quantum yield of PSII photochemistry under light of different PAR intensities (Φ_PSII_) was calculated as (*F*_m_′−*F*_t_)/*F*_m_′. The basal fluorescence of light-adapted leaves was recorded after rapid reoxidation of the PQ pool using far-red light. The fraction of PSII centers that are closed under light of different PAR intensities, also known as excitation pressure (1-qP) was calculated as (*F*_t_−*F*_0_′)/*F*_m_′−*F*_0_^′^). The non-photochemical quenching of Chl fluorescence (*NPQ*) was calculated using the equation *NPQ* = (*F*_m_−*F*_m_′)/*F*_m_′.

### EPR spectroscopy

Room temperature EPR measurements were performed on isolated thylakoid membranes placed in a flat cell with a Bruker ELEXYS E500 spectrometer equipped with a SuperX EPR049 microwave bridge and a SHQ4122 cavity. Measurements were performed in thylakoid membranes placed in the flat cell. PSI/PSII ratio was determined from the EPR signals from PSII (Tyr_D_ **^•^** radical) and from PSI (P_700_^+^ radical, in the presence of 10 mM ferricyanide) as described by Danielsson et al. [51].

### Quantitative analysis of PSII protein phosphorylation by mass spectrometry

For the quantitative mass spectrometry studies the thylakoids isolated from the Col-0, Ws-4 or L*er*-0 plants were resuspended in 25 mM NH_4_HCO_3_, 10 mM NaF to a final concentration of 2.5 mg Chl/ml and incubated for 3 h at 22°C with sequencing grade-modified trypsin (Promega) at 5 mg enzyme/mg Chl [52], [53]. Released peptides were separated from the thylakoid membranes by 30 min centrifugation at 100 000 g. Peptides were analyzed using an on-line nano-flow HPLC system (EASY-nLC; Proxeon, Bruker Daltonics) in conjugation with a ion trap mass spectrometer HCTultra PTM Discovery System (Bruker Daltonics). A 20 mm×100 µm pre-column followed by a 100 mm×75 µm analytical column (Nano Separations) both packed with reverse-phase C18 were used for separation at a flow rate of 300 nL/min. The LC gradient buffers used were A = 0.1% formic acid in water and B = 0.1% formic acid in 100% acetonitrile and separation was performed for 240 min as follow: 0–15% B in first 110 min; 15%–40% B in 110–200 min; 40%–100% B in 200–220 min and 100% B in 220–240 min. Automated online tandem MS/MS analyses were performed using collision induced dissociation of peptide ions. The proteolytic shaving of the surface-exposed phosphorylated protein domains has proven to be very efficient to remove all the protein parts extending out of the membrane, and thus releasing both the phosphorylated and the non-phosphorylated parts of a protein. To make a quantitative estimation of the PSII phosphorylation between the accessions we used the recently described normalization method [4].

### Electron microscopy

Thylakoid ultrastructure was visualized by electron microscopy essentially as previously described [13]. From all samples at least 50 different plastids were studied (chosen randomly from at least 100 plastids plastids on 5–6 different ultrathin sections from the cross sections of two different leaves) and representative pictures are shown. Calculations of plastid size and number of grana *per* plastid section were done on 50–80 representative plastids. The granum height and width (diameter) were calculated from analyzing 250 to 657 grana in each accession (for data, see Table S2). The granum height and width were defined as the number of appressed thylakoid lamellae and the width (diameter) at the middle of the granum, respectively.

### Photoinhibitory treatment

For photoinhibition experiments, detached leaves were treated with 2 mM lincomycin in darkness overnight. The leaves together with the 2 mM lincomycin were then transferred to Petri dishes and exposed to HL (950 µmol photons m^−2^ s^−1^) for 3 h at 22°C. Chl fluorescence parameter *F*_v_/*F*_m_ was measured using PAM-210 in control and lincomycin-treated leaves after 5 min of dark adaptation.

### SDS-PAGE, BN-PAGE and western blotting

SDS-polyacrylamide gel electrophoresis (SDS-PAGE), Blue-native PAGE and western blotting were performed as described [13]. Antibodies against the D1, CP43, CP29, Lhcb1, Lhcb2 subunits of PSII, the PsaB subunit of PSI, the CF1 *β*-subunit of ATP-synthase, the Cytf subunit of Cytb_6_f complex, STN7 and STN8 proteins were purchased from Agrisera (Umeå, Sweden). Where indicated, rabbit anti-phosphothreonine antibodies from Cell Signaling (New England BioLabs) and Zymed (Invitrogen) were used. The immunodetection was performed using ECL system (GE Healthcare), visualized using Fuji-4000 and quantified using Multi-Gauge system (Fuji, Japan).

### Statistical analysis

The mean and standard deviation were calculated for each data set where appropriate. Standard deviation bars were plotted except where smaller than the symbol size. Where appropriate, the Student's t-test was used to identify the difference between the three accessions. In case of several parameters related to plastid ultrastructure, the data did not follow normal distribution, therefore, the non-parametric methods Mann-Whitney U-test *per* data pairs and Kruskal-Wallis tests have been used. Advanced statistical analyses have been made with the software GraphPad InStat (GraphPad Software Inc., La Jolla, USA).

## Supporting Information

Figure S1 Photographs of representative Ws-4, Col-0 and L*er*-0 *Arabidopsis* plants grown on soil for six weeks at an irradiance of 120 µmol photons m^−2^ s^−1^.
(TIF)

Figure S2 Fast Chl *a* fluorescence induction curves of Col-0, Ws-4 and L*er*-0 *Arabidopsis* accessions.
(TIF)

Figure S3 The xanthophyll pigment composition in L*er*-0, Col-0 and Ws-4 accessions.
(TIF)

Table S1 Leaf biomass and chlorophyll content of Col-0, Ws-4 and L*er*-0 accessions grown on soil.
(PDF)

Table S2 Parameters of chloroplast ultrastructure in Col-0, Ws-4 and L*er*-0 accessions.
(PDF)

Table S3 Thylakoid lipid content of leaf tissue from Col-0, Ws-4 and L*er*-0 accessions.
(PDF)

Table S4 Relative amounts of various types of PSII complexes in Col-0, Ws-4, and L*er*-0 accessions.
(PDF)
