# Supplementary figures and images for: Photosystem II Function and Dynamics in Three Widely Used Arabidopsis thaliana Accessions
Source: PLoS One. 2012 Sep 28;7(9):e46206. doi: 10.1371/journal.pone.0046206 (PMC3460815; doi:10.1371/journal.pone.0046206)

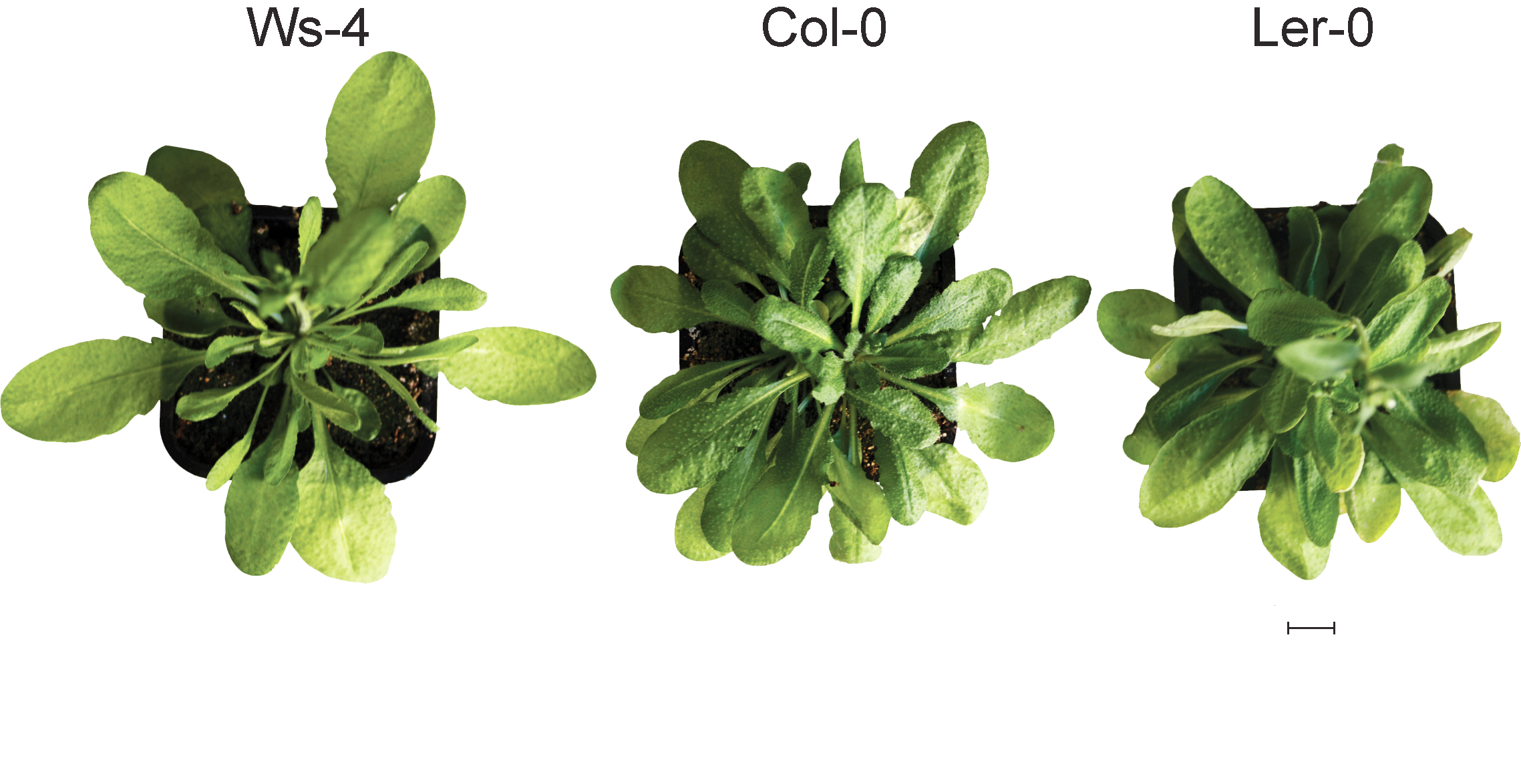

Supplement: Figure S1 — Photographs of representative Ws-4, Col-0 and Ler-0 Arabidopsis plants grown on soil for six weeks at an irradiance of 120 µmol photons m−2 s−1. (TIF) [file pone.0046206.s001.tif]

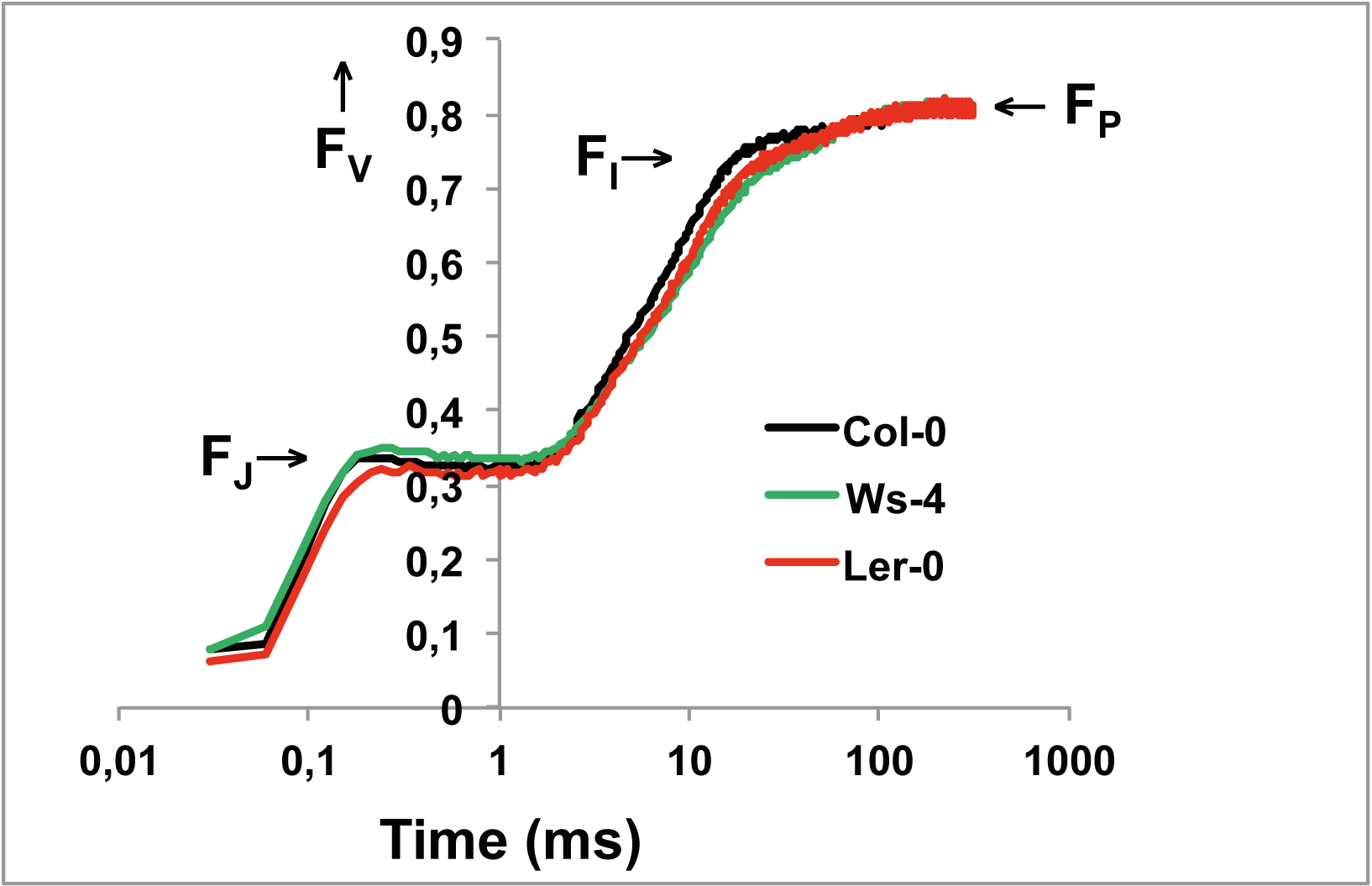

Supplement: Figure S2 — Fast Chl a fluorescence induction curves of Col-0, Ws-4 and Ler-0 Arabidopsis accessions. (TIF) [file pone.0046206.s002.tif]

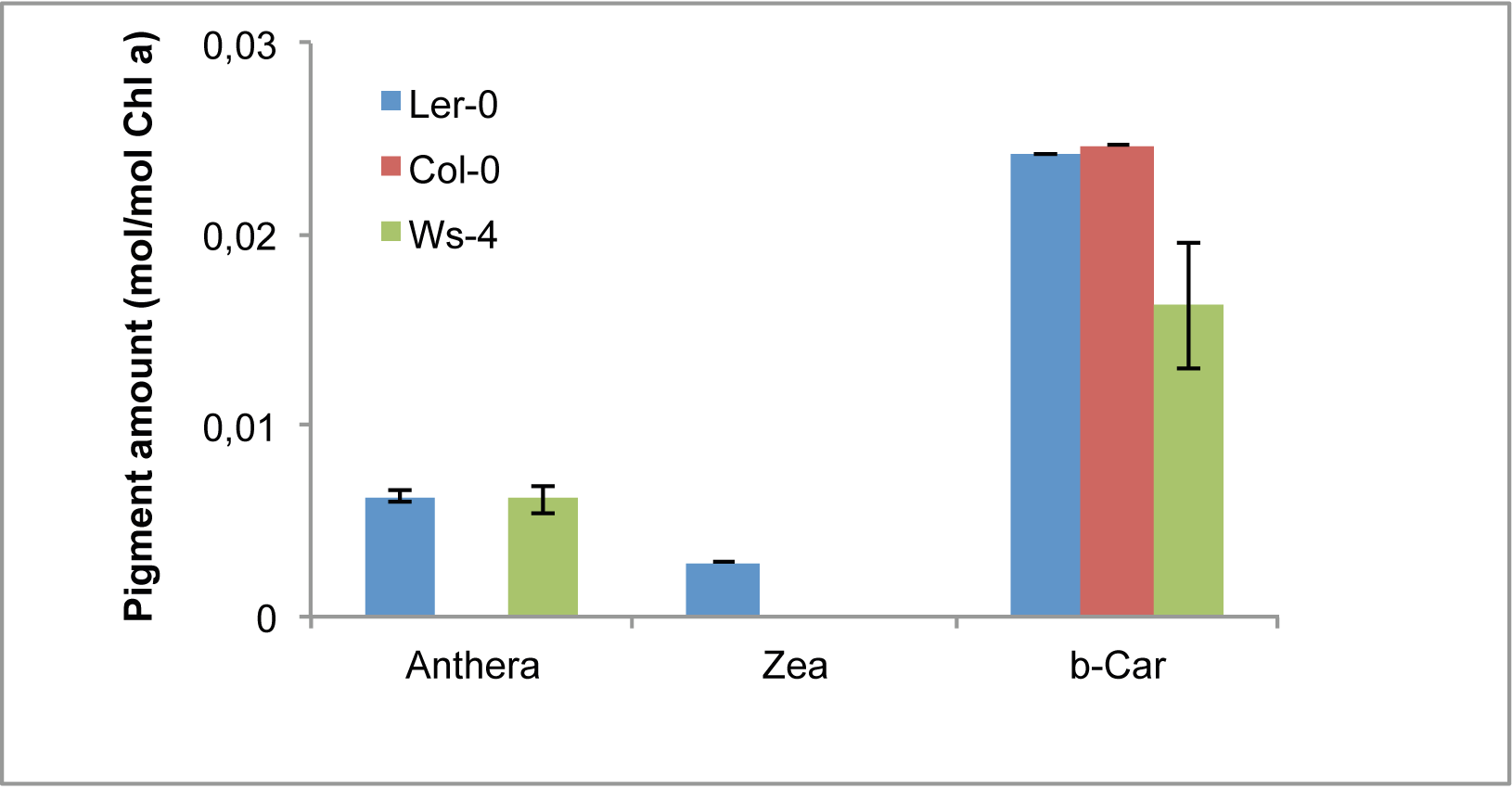

Supplement: Figure S3 — The xanthophyll pigment composition in Ler-0, Col-0 and Ws-4 accessions. (TIF) [file pone.0046206.s003.tif]
