# Supplementary material for: Photosystem II Function and Dynamics in Three Widely Used Arabidopsis thaliana Accessions
Source: PLoS One. 2012 Sep 28;7(9):e46206. doi: 10.1371/journal.pone.0046206 (PMC3460815; doi:10.1371/journal.pone.0046206)
Supplement: Table S1 — Leaf biomass and chlorophyll content of Col-0, Ws-4 and Ler-0 accessions grown on soil. (PDF) [file pone.0046206.s004.pdf]

Table S1. Leaf biomass and chlorophyll content of Col-0, Ws-4 and L*er*-0 accessions grown on soil. The parameters were measured on plants grown for six weeks on soil at an irradiance of 120 µmol photons m^{-2} s^{-1} and were expressed as means ±SD (n=number of replicates). The parameters are also expressed relative to Col-0. *, Significantly different from Col-0 (Student's t-test P<0.05).

**Accession**	**Shoot fresh weight**	**Leaf specific mass**	**Leaf chlorophyll content**[a]		
	g plant^{-1} *(n=16)*	*mg cm*$^{-2}$ *(n=8)*	*µg cm*$^{-2}$ *(n=8)*	*mg g*$^{-1}$ *(n=8)*	*Chl a/b (n=8)*
Col-0	1.59±0.32<br>100%	17.95±0.99<br>100%	19.93±2.30<br>100%	1.11±0.14<br>100%	3.93±0.10
Ws-4	1.51±0.32<br>95%	13.82±1.05*<br>77%	15.35±3.68*<br>77%	1.13±0.30<br>101%	3.93±0.11
L*er*-0	1.35±0.33*<br>85%	16.86±1.01<br>94%	20.12±1.73<br>101%	1.19±0.10<br>107%	3.78±0.11

[a]Leaf chlorophyll content was measured spectrophotometrically after extraction in ethanol.
