# Supplementary material for: Photosystem II Function and Dynamics in Three Widely Used Arabidopsis thaliana Accessions
Source: PLoS One. 2012 Sep 28;7(9):e46206. doi: 10.1371/journal.pone.0046206 (PMC3460815; doi:10.1371/journal.pone.0046206)
Supplement: Table S2 — Parameters of chloroplast ultrastructure in Col-0, Ws-4 and Ler-0 accessions. (PDF) [file pone.0046206.s005.pdf]

Table S2. Parameters of chloroplast ultrastructure in Col-0, Ws-4 and L*er*-0 *Arabidopsis* accessions. The plants were grown hydroponically for 4 weeks at an irradiance of 120 µmol photons m^{-2} s^{-1} (GL). HL samples originated from GL plants subsequently illuminated for 3 h with high-intensity light (HL=950 µmol photons m^{-2} s^{-1}).

**Parameter**	**Col-0**	**Ws-4**	**L*er*-0**
**GL (120 µmol photons m^{-2} s^{-1})**			
Plastid length (µm)	6.2 ± 1.0	6.1 ± 1.2	6.6 ± 1.3*
Plastid width (µm)	3.6 ± 0.6	3.5 ± 0.6	3.2 ± 0.7*
Plastid width/length	0.6 ± 0.1	0.6 ± 0.1	0.5 ± 0.1*
Starch/plastid section	3.8 ± 1.7*	3.0 ± 1.5	2.5 ± 1.8
Grana/plastid section	60 ± 11*	67 ± 14	70 ± 15
Grana height	6.2 ± 3.2	5.8 ± 3.4	6.0 ± 3.9
Grana width (µm)	0.53 ± 0.18**	0.55 ± 0.20	0.56 ± 0.23**
**HL (950 µmol photons m^{-2} s^{-1})**			
Plastid length (µm)	6.8 ± 1.2	6.3 ± 1.0**	7.0 ± 1.3**
Plastid width (µm)	3.8 ± 0.7	4.2 ± 0.7*	3.7 ± 0.9
Plastid width/length	0.6 ± 0.1	0.7 ± 0.1*	0.5 ± 0.2
Starch/plastid section	3.7 ± 1.9	3.2 ± 1.9	3.2 ± 2.0
Grana/plastid section	55 ± 10**	57 ± 12	62 ± 15**
Grana height	6.0 ± 3.3	5.1 ± 2.6*	6.3 ± 4.0
Grana width (µm)	0.54 ± 0.15	0.51 ± 0.15*	0.54 ± 0.17

The presented data were determined based on electron micrographs similar to those shown in Figures 1 and 10. Statistical analysis was performed for 50-80 plastids from each accession for most parameters, and the mean values ±SD were presented. Data about grana height (number of appressed thylakoid lamellae) and width (defined at the middle of the granum) were determined from measurements on 250-657 grana from 50-80 representative plastids from 5-6 ultrathin sections from the cross sections of two different leaves of each type of treated sample. Student's t-test was used for the analyses of most of the ultrastructural data. However, when the data obtained (*e.g.* for grana height and length) failed normality analysis, the non-parametric method Mann-Whitney U-test per data pairs and Kruskal-Wallis test were used. Significant differences are indicated with ** between two different samples. *, Significantly different in the indicated accession from the other two accessions ($P < 0.05$).
