# Supplementary material for: Photosystem II Function and Dynamics in Three Widely Used Arabidopsis thaliana Accessions
Source: PLoS One. 2012 Sep 28;7(9):e46206. doi: 10.1371/journal.pone.0046206 (PMC3460815; doi:10.1371/journal.pone.0046206)
Supplement: Table S3 — Thylakoid lipid content of leaf tissue from Col-0, Ws-4 and Ler-0 accessions. (PDF) [file pone.0046206.s006.pdf]

Table S3. Thylakoid lipid content of leaf tissue from Col-0, Ws-4 and L*er*-0 accessions. Lipids were extracted from leaves harvested from plants grown on soil for six weeks at an irradiance of 120 μmol photons m^{-2} s^{-1}.

**Accession**	**MGDG**		**DGDG**		**SQDG**		**PG**	
	μmol g⁻¹	*nmol cm⁻²*	*μmol g⁻¹*	*nmol cm⁻²*	*μmol g⁻¹*	*nmol cm⁻²*	*μmol g⁻¹*	*nmol cm⁻²*
Col-0	1.82±0.07	32.67±0.80	0.97±0.05	17.41±0.60	0.20±0.01	3.59±0.20	0.70±0.05	12.56±0.70
	100%	100%	100%	100%	100%	100%	100%	100%
Ws-4	2.37±0.08*	32.75±0.60	1.36±0.08*	18.79±0.70	0.23±0.01	3.18±0.10	0.90±0.10	12.43±0.80
	130%	100%	140%	108%	115%	89%	129%	99%
L*er*-0	1.74±0.01	29.33±0.10	1.04±0.01	17.53±0.10	0.19±0.01	3.20±0.10	0.67±0.02	11.29±0.30
	96%	90%	107%	100%	95%	89%	96%	90%

The lipid content was expressed as means ±SD (n=3) on a leaf fresh weight and leaf area basis. The lipid content was also expressed relative to Col-0. *, Significantly different from Col-0 (Student's t-test P<0.05).
