# Supplementary material for: Photosystem II Function and Dynamics in Three Widely Used Arabidopsis thaliana Accessions
Source: PLoS One. 2012 Sep 28;7(9):e46206. doi: 10.1371/journal.pone.0046206 (PMC3460815; doi:10.1371/journal.pone.0046206)
Supplement: Table S4 — Relative amounts of various types of PSII complexes in Col-0, Ws-4, and Ler-0 accessions. (PDF) [file pone.0046206.s007.pdf]

Table S4. Relative amounts of various types of PSII complexes in Col-0, Ws-4, and L*er*-0 accessions. The plants were grown hydroponically for six weeks at an irradiance of 120 µmol photons m^{-2} s^{-1} and treated for 3 h at an irradiance of 950 µmol photons m^{-2} s^{-1} (high light) in the absence or presence of lincomycin (LN). Thylakoid membranes were isolated and solubilized mildly with n-dodecyl-β-D-maltoside, and the various types of Chl protein complexes were separated by Blue-native gel electrophoresis. As dark control, 16 h dark-adapted plants were used.

Dark-adapted	Col-0	Ws-4	L*er*-0
PSII-LHCII supercomplex	20±2	20±2	32±5
PSII core dimer	13±1	10±1	10±2
PSII core monomer	40±2	39±2	36±1
PSII core CP43-less monomer	27±1	31±1	22±1
PSII monomer : PSII dimer	2:1	2:1	1.5:1
High light - LN	Col-0	Ws-4	L*er*-0
PSII-LHCII supercomplex	7±2	4±2	7±1
PSII core dimer	14±2	9±1	9±1
PSII core monomer	35±5	40±3	36±2
PSII core CP43-less monomer	44±5	47±1	48±3
PSII monomer : PSII dimer	4:1	7:1	5:1
High light + LN	Col-0	Ws-4	L*er*-0
PSII-LHCII supercomplex	5±2	1±0*	4±2
PSII core dimer	15±2	4±2*	8±3
PSII core monomer	35±3	40±3	41±3
PSII core CP43-less monomer	45±3	55±3*	46±3
PSII monomer : PSII dimer	4:1	20:1	7:1

The relative amounts of four types of PSII complexes were determined from the quantification of D1 protein in western blots of Blue-native gels as in Figure 11, and the ratio of PSII monomer-to-dimer was calculated for each accession and condition. Data represent means ±SD of three independent preparations. *, Significantly different from Col-0 and L*er*-0 (Student's t-test $P < 0.01$).
